# Author Correction: Metal-coordinated sub-10 nm membranes for water purification

**DOI:** 10.1038/s41467-019-12959-z

**Published:** 2019-11-08

**Authors:** Xinda You, Hong Wu, Runnan Zhang, Yanlei Su, Li Cao, Qianqian Yu, Jinqiu Yuan, Ke Xiao, Mingrui He, Zhongyi Jiang

**Affiliations:** 1 Key Laboratory for Green Chemical Technology, School of Chemical Engineering and Technology, Tianjin University, Tianjin, 300072 China; 2 Collaborative Innovation Center of Chemical Science and Engineering (Tianjin), Tianjin, 300072 China; 3 Tianjin Key Laboratory of Membrane Science and Desalination Technology, Tianjin University, Tianjin, 300072 China

**Keywords:** Membrane structure and assembly, Environmental sciences, Coordination chemistry

Correction to: *Nature Communications* 10.1038/s41467-019-12100-0, published online 12 September 2019.

The original version of this Article contained errors in Fig. 4 and Supplementary Fig. 5. In Fig. 4a, some of the permeance data in each of the three solutions were inadvertently incorrectly transferred from the original data sheets to the plotting software.

The correct version of Fig. 4a is

The incorrect original version of Fig. 4a is



This has been corrected in both the PDF and HTML versions of the Article.

In Supplementary Fig. 5, the SEM image of MOPM-Ni^2+^ was inadvertently replaced by a duplicate of MOPM-Zn^2+^. The HTML has been updated to include a corrected version of the Supplementary Information.

