# Author Correction: Metal-coordinated sub-10 nm membranes for water purification

**DOI:** 10.1038/s41467-020-14339-4

**Published:** 2020-01-24

**Authors:** Xinda You, Hong Wu, Runnan Zhang, Yanlei Su, Li Cao, Qianqian Yu, Jinqiu Yuan, Ke Xiao, Mingrui He, Zhongyi Jiang

**Affiliations:** 1 Key Laboratory for Green Chemical Technology, School of Chemical Engineering and Technology, Tianjin University, Tianjin, 300072 China; 2 Collaborative Innovation Center of Chemical Science and Engineering (Tianjin), Tianjin, 300072 China; 3 Tianjin Key Laboratory of Membrane Science and Desalination Technology, Tianjin University, Tianjin, 300072 China

**Keywords:** Membrane structure and assembly, Environmental sciences, Coordination chemistry

Correction to: *Nature Communications* 10.1038/s41467-019-12100-0, published online 08 November 2019.

The original version of this Article contained errors in Fig. 3. In Fig. 3e, the permeance data of the ‘5-layers’ and ‘7-layers’ membranes were inadvertently incorrectly transferred from the original data sheets to the plotting software.

The original version of this Article also contained errors in the second and third sentences of the first paragraph of the ‘Structural optimization of ultrathin MOPMs’ section of the Results, which incorrectly read ‘As the PA concentration was reduced from 0.045 to 0.030 mg/mL, the thickness of the resultant membrane decreased from 21.2 ± 0.2 to 13.2 ± 0.7 nm (Supplementary Figs. 7 and 8). By further decreasing the PA concentration to 0.015 mg/mL, a sub-10 nm membrane was achieved (Fig. 2a)’. The correct version states ‘4.5–3.0 mg/mL’ in place of ‘0.045–0.030 mg/mL’, and ‘1.5 mg/mL’ instead of ‘0.015 mg/mL’.

The tenth sentence in the legend of Fig. 2 originally incorrectly read ‘For **a–c**, the PA concentration was 0.015 mg/mL’. The correct version reads ‘1.5 mg/mL’ instead of ‘0.015 mg/mL’.

The fourth sentence of the first paragraph of the Discussion originally incorrectly read ‘Impressively, we tuned the MOPM-Fe^3+^ membrane in thickness to 8-nm through reducing the ligand concentration to 0.015 mg/mL’. The correct version states ‘1.5 mg/mL’ instead of ‘0.015 mg/mL’.

These have been corrected in both the PDF and HTML versions of the Article.

Similarly, the original version of the Supplementary Information associated with this Article contained errors throughout in which the correct PA concentrations of ‘1.5 mg/mL’, ‘3.0 mg/mL’ and ‘4.5 mg/mL’ where incorrectly given as ‘0.015 mg/mL’, ‘0.030 mg/mL’ and ‘0.045 mg/mL’, respectively. The HTML has been updated to include a corrected version of the Supplementary Information where this error has been fixed in Supplementary Fig. 7 and its legend, Supplementary Fig. 8 and its legend, the legend of Supplementary Figs. 11–13, the legend of Supplementary Figs. 16–24, the legend of Supplementary Fig. 26 and in the ‘PA concentration (mg/mL)’ column of Supplementary Table 7.

