# Supplementary material for: Metal-coordinated sub-10 nm membranes for water purification
Source: Nat Commun. 2019 Sep 13;10:4160. doi: 10.1038/s41467-019-12100-0 (PMC6744495; doi:10.1038/s41467-019-12100-0)
Supplement: Supplementary file 1 — Supplementary Information [file 41467_2019_12100_MOESM1_ESM.pdf]

Supplementary Information

Metal-coordinated sub-10 nm membranes for water purification

You et al.

Supplementary Methods

Materials

All reagents and solvents were commercially available and used as received. Phytic acid (PA, 50 wt% in water) was purchased from Heowns Co. (Tianjin, China). Silver nitrate ($AgNO_3$), zinc chloride ($ZnCl_2$), nickel chloride hexahydrate ($NiCl_2 \cdot 6H_2O$), ferric chloride hexahydrate ($FeCl_3 \cdot 6H_2O$), zirconium nitrate pentahydrate ($Zr(NO_3)_4 \cdot 5H_2O$) were obtained from Kemiou Chemical Reagent Co. (Tianjin, China). Methyl blue, Congo red, Alcian blue, Rose Bengal and Orange GII were purchased from Aladdin Industrial Co. (Shanghai, China). Poly(ethylene glycol) (PEG, M_w=200, 400, 600, 1000, 2000, 4000, 6000 Da), hydrochloric acid (HCl), sodium hypochlorite (NaClO), sodium hydroxide (NaOH), sodium chloride (NaCl), sodium sulfate (Na_2SO_4), magnesium sulfate ($MgSO_4$), magnesium chloride ($MgCl_2$), sodium dihydrogen phosphate (NaH_2PO_4) and disodium hydrogen phosphate (Na_2HPO_4) were purchased from Jiangtian Chemical Reagent Co. Bovine serum albumin (BSA), humic acid (HA), sodium alginate (SA) and lysozyme as model foulants were purchased from Institute of Hematology, Chinese Academy of Medical Science (Tianjin, China), Kewei Chemical Reagent Co. (Tianjin, China) and Yuanli Chemical Reagent Co. (Tianjin, China), respectively. Partially-hydrolyzed polyacrylonitrile porous substrates (PAN, MWCO=100 kDa) were supplied by Lanjing Membrane Technology Co. (Shandong, China). Water used in all experiments was prepared by a Milli-Q ultrapure unit (pH =6.0 ±0.2).

Assembly of MOPMs

Firstly, the commercial PAN substrate (tailored into circle with diameter of 5 cm) was immersed in ethanol followed by air drying to remove contaminant. Secondly, the PAN substrate was facedown submerged in PA solution (25.5 mL) in 300 mL beaker with varied PA concentration for 5 min. Sequently, 4.5 mL of metal salt solution with varied metal salt content was poured into PA solution to trigger PA assembly for a schedule time. Afterwards, the assembly solution with PAN substrate coated by MOPMs was integrally transferred into oven for thermal curing at 60 ^{o}C for 10 min. Finally, the MOPM/PAN composite membrane was rinsed with DI water (pH=6.0±0.2) for 10 min to remove most residual weakly bound PA molecules and metal ions and stored in DI water before use. The resultant membrane was denoted as MOPM-M^{n+}, where the M^{n+} represented Ag^{+}, Zn^{2+}, Ni^{2+}, Fe^{3+} and Zr^{4+}.

Characterization

All the membrane samples were freeze-dried overnight before characterization.

SEM. Field emission scanning electron microscopy (Nanosem 430, Japan) was utilized to capture the surface and cross-section morphology of membrane. The Au coating was sputtered on samples surface (Q150T turbo-pumped sputter coater) with current of 25 mA for 1 min at argon atmosphere (2×10^{-2} mbar) to achieve a minimum conductivity and avoid sample charging.

TEM. Transmission electron microscopy (JEM-2100F, Japan) was employed to capture the cross-section image of membrane. The membrane sample was encapsulated by epoxy resin and cut into thin (~90–100 nm) slices by Ultratome (Leica EM UC6). Finally, the slice-shaped samples were deposited on micro grid copper.

AFM. Atomic force microscopy (Bruker Dimension Icon, USA) was employed to characterize the surface roughness and thickness of membranes. The images were captured using tapping mode. NanoscopeAnalysis data visualization and analysis software was used to process the AFM images. Surface roughness was presented by root-mean-square (R_{rms}). Surface morphology, roughness parameters and thickness were evaluated from AFM scans. To measure the thickness, the MOPMs was assembled on quartz glass wafers. A scratch was made with a sharp AFM probe[1] with approximately 2 nN applied force. Membrane thickness was detected from the height difference between glass substrate and the membrane using a one dimensional statistical function. The surface strength of the membranes was expressed by Young's modulus (E), and calculated using (Eq. 1)

$$z = d + \sqrt{\frac{k}{(\pi/2)\left[E/\left(1-v^2\right)\right]\tan\alpha}}\sqrt{d} \tag{1}$$

where E was the Young’s modulus, d and z were the cantilever deflection and piezo displacement, respectively. k was the spring constant of the cantilever (k = 39 N/m), v was Poisson’s ratio (v = 0.5), and α is the opening angle of the cone (α = 35°). AFM results were analyzed and exported by bundled software (NanoScopeAnalysis Version1.9, Bruker Dimension Icon).

EDX. Energy dispersive X-ray spectroscopy (Genesis XM2 APEX 60SEM, USA) was utilized to achieve the elemental distribution and composition of membranes. The EDS detector was externally affiliated to the SEM apparatus and the EDS mapping images and elemental ratio could be obtained under similar condition of SEM characterization.

ATR-FTIR spectra. Attenuated total reflectance fourier transform infrared spectroscopy (Nicolet 560, USA) was utilized to characterize the chemical structures of MOPMs.

CA. Contact angle goniometer (Data-Physics OCA 15EC, China) was utilized to measure the sessile drop contact angles of DI water on the membrane samples. The instantaneous CA value was capture by high-speed camera. The dynamic CA value was collected by continuous shooting.

UV-vis spectra. Ultraviolet-visible spectrophotometer (Hitach UV-3010, Japan) was applied to analyze the dye concentration and coordination between PA and Fe^{3+}. For dye concentration, according to Lambert-Beer's Law, the linear correlation between solute concentration (*C*) and absorbance (*A*) was reliable when *A* was in range of 0.2-0.8. Notably, the *A* value of 100 ppm and 50 ppm dye feed was beyond the prescribed scope (approximately in range of 1-3), which should be diluted 10:1 or 5:1 for measurement. Therefore, the exact dye rejection (%) of membranes with different feed (100, 50 and 10 ppm) could be calculated by the *A* of diluted dye solution (10 ppm) and permeate at maximum absorption wavelength of dye by following equation (Eq. 2):

$$R=\frac{C_f - C_p}{C_f}\times 100\%=\frac{10\times A_{f(10\ \mathrm{ppm})} - A_p}{10\times A_{f(10\ \mathrm{ppm})}}\times 100\% \quad \text{or} \quad \frac{5\times A_{f(10\ \mathrm{ppm})} - A_p}{5\times A_{f(10\ \mathrm{ppm})}}\times 100\% \quad \text{or} \quad \frac{A_{f(10\ \mathrm{ppm})} - A_p}{A_{f(10\ \mathrm{ppm})}}\times 100\% \qquad (2)$$

where C_p (ppm) and C_f (ppm) were the solute concentration in permeate and feed solutions, respectively. The maximum absorption wavelength of Methyl blue, Congo red, Alcian blue, Rose Bengal and Orange GII were 590 nm, 496 nm, 600 nm, 506 nm and 476 nm, respectively.

Raman spectra. DXR Smart Raman spectrometer (Thermo Fisher Scientific, USA) was employed to characterize the coordination interaction of MOPM-Fe^{3+}. The PAN substrate suffered from severe fluorescence interference to cover the characteristic peaks. In this regard, the quartz glass wafer replaced the PAN as substrate to obtain clear Raman spectra. The glass substrates were immersed in $FeCl_3$ and PA solution to obtain Raman spectra of Fe^{3+} and PA. The Raman spectroscopy was acquired by a He–Ne laser at a wavelength of 633 nm.

DLS. Dynamic light scattering (Zetasizer nano ZS90, UK) was utilized to measure the size of Fe^{3+}-PA complex and the chargeability of dyes. The assembly time of Fe^{3+}-PA complex was fixed at 60 min. The samples were pre-treated with ultrasonic for 30 min at 100% power before measurement.

XPS. X-ray photoelectron spectroscopy (ESCALAB 250Xi, USA) was employed to analyze the element components by using Al Kα (1486.6 eV) as the radiation source. Data processing and deconvolution of high-resolution scan spectra were performed in XPSPEAK41 software. Peak area was measured after background subtraction following the methods of Shirley. The deconvolution was based on the targeted peak positions in reported references. For accuracy of deconvolution, the Lorentzian-Gaussian (L/G) were confined around 20% and the full width at half maximum (FWHM) was not exceeding 2.7 eV.

Zeta potential. SurPASS Electrokinetic Analyzer (Anton Paar KG, Austria) was employed to investigate the surface zeta potential of membranes. A rectangle clamp cell was utilized to fix the membrane samples. For each measurement, membrane sample was tailored into sheet (1×2 cm^2) and attached onto a holder with double-sided water-resistant tape, followed by fixing the holder into the cell. The system was cleaned with DI water before each

test. The conductivity was calibrated prior to each test. To measure isoelectric point of PAN substrate, HCl (0.1 mol/L) and NaOH (0.1 mol/L) were used as titration solution.

Pore size distribution. The pore size distribution of membranes was measured *via* rejection experiments using PEG with different molecular weight (M_w=200, 400, 600, 1000, 2000, 4000, 6000 Da) at concentration of 50 ppm as feed solution[2]. The concentrations of permeate and feed solutions were determined by a chemical oxygen demand analyzer. The Stokes radii of PEG was calculated based on their average molecular weight by following equation (Eq. 3),

$$r = 16.73 \times 10^{-12} \times M_w^{\ 0.557} \tag{3}$$

where r (m) was the Stokes radii of PEG and Mw was the average molecular weight (Da). Subsequently, we related obtained solute rejection with the Stokes radii and transformed it into a correlation function. Finally, the pore size distribution was described by the following probability density function namely Eq. 4,

$$\frac{\mathrm{d}R\left(d_p\right)}{\mathrm{d}d_p} = \frac{1}{d_p \ln\sigma_p \sqrt{2\pi}} \exp\left(-\frac{\left(\ln d_p - \ln\mu_p\right)^2}{2\left(\ln\sigma_p\right)^2}\right) \tag{4}$$

where μ_p was defined as the geometric mean diameter of solute at 50% solute rejection, σ_p was defined as the ratio of the solute radius when solute rejections were 84.13% and 50%, representing the geometric standard deviation of μ_p.

Filtration performance measurement

The membrane was loaded in the filtration cell with effective area of 4.1 cm^2 and maximum volume of 10 mL. Before measurement, the membrane was prepressed with DI water at 1.5 bar for 30 min to obtain steady flux. Then, the filtration performance of membrane was measured at 1.0 bar with 10 ppm, 50 ppm and 100 ppm of dye solution as feed (Methyl blue, Congo red, Alcian blue, Rose Bengal and Orange GII). Especially, the graphene oxide quantum dots (GQDs) were synthesized based on our previous reported work[3] and used as filtration feed (1wt%) to simulated nanoadsorbents. Besides, the salt permeation was also measured by rejecting 1000 ppm of salt solution feed (NaCl, $NaSO_4$, $MgSO_4$, $MgCl_2$), which was calculated by the conductivity of feed and permeate with a conductivity analyzer (Leichi, DDS-11A, China) using following equation (Eq. 5),

$$P = \frac{C_p}{C_f} \times 100\% \tag{5}$$

where the C_p and C_f were the salt concentration in permeate and feed solutions. The dye adsorption was measured

by fixing membrane into filtration cell (effective area=1.77 cm^2) with 10 mL of feed solution (100 ppm dye). After 3-day adsorption, the dye concentration of feed was determined by ultraviolet-visible spectrophotometer and the amount of adsorbed dyes (M_{dye}, $\mu g/cm^2$) on the membrane was calculated by following equation (Eq. 6)

$$M_{dye} = \frac{V_S}{A} \times (C_0 - C_3) \quad (6)$$

where the C_0, C_3 (μg/mL) were the dye concentration at the beginning and after 3 days, respectively, V_S (mL) was the volume of solution, the A (cm^2) was the effective membrane area. The reported data were the mean values of triplicate samples for each membrane.

Stability performance measurement

For antifouling performance, the MOPM-Fe^{3+}/PAN composite membrane was pre-compacted at 1.5 bar for 30 min for stabilized water permeation flux. The concentration of SA, HA, BSA and lysozyme were all 1000 ppm. The phosphate buffer solution (0.1 M, pH=6.0) composed of NaH_2PO_4 and Na_2HPO_4 was used to prepare BSA feed[4]. Firstly, the membrane was tested with DI water at 1.0 bar for 30 min to get initial permeance (J_{n-1}). Secondly, filtration process was carried out for 30 min with feed solution containing 1000 ppm foulant to get filtration permeance ($J_{(n-1)f}$). Finally, after rinsing membrane with DI water by magnetic stirring at 200 rpm for 5 min, recovered permeance (J_n) of membrane was measured at 1.0 bar for 30 min. The as-described filtration-rinse-test process was repeated five times and the permeance was normalized for comparison. The n (n=1,2,3,4,5) represented filtration cycle. Permeance recovery ratio ($P_{RR}= J_n/J_{n-1}$) and permeance decline ratio ($P_{DR}=1- J_{(n-1)f}/J_{n-1}$) were calculated to evaluate the antifouling performance of membrane for each cycle.

The 5-day stability performance of membranes was carried out by filling DI water into buffer tank to supply enough feed. Owing to the nearly 100% Congo red rejection of MOPM-Fe^{3+}/PAN composite membrane, it was reasonable to assume there was no dye loss in feed and maintain a constant feed concentration (100 ppm) during long-term performance measurement.

The BSA adsorption measurement was conducted with method we previous reported[4]. Firstly, the membrane samples were tailored into circle sheets (diameter =4.5 cm) rinsed with phosphate buffer (0.1 M, pH=6.0) then wiped with a piece of filter paper to remove the water on the surface. Secondly, the samples were fixed into filtration cell (Amicon 8200) with 20 mL BSA solution (1000 ppm). The static and dynamic adsorption measurements were carried out under the unstirred condition and stirred condition (200 rpm), respectively. After incubation for 12 h at room temperature (25 °C) to reach adsorption–desorption equilibrium, the concentrations of

BSA in the solution before and after incubation were measured with a UV–vis spectrophotometer, and then the amount of adsorbed BSA (M, μg/cm^2) on the membrane was calculated by following equation (Eq. 7),

$$M=\frac{V_S}{A}\times(C_0\text{-}C_1) \tag{7}$$

where the C_0, C_1 (μg/mL) were the BSA concentration at the beginning and adsorption equilibrium (1 day), respectively, V_S (mL) was the volume of solution, the A (cm^2) was the membrane area. The reported data were the mean values of triplicate samples for each membrane. The BSA concentration was determined by UV-vis spectrophotometer at 278 nm.

The stability of MOPM-Fe^{3+}/PAN membrane in salty condition was conducted by immersing membrane in Na_2SO_4 solution (1000 ppm) or NaCl solution (1000 ppm) followed by performance measurement every two days. This immersion test was carried out for 2 weeks.

The metal ion leakage of the five types of as-prepared MOPM/PAN membranes was measured by detecting metal element (Ag^+, Zn^{2+}, Ni^{2+}, Fe^{3+} and Zr^{4+}) in filtrated water. 30 mL DI water was filtrated through MOPM/PAN membranes and collected for metal element content (ppm) measurement by inductively coupled plasma (ICP, Leeman Prodigy, USA).

The Arrhenius equation,

$$k = Ae^{(-E_a/RT)} \tag{8}$$

where k is the solvent permeation speed (L m^{-2} h^{-1}), A is the pre-exponential factor (L m^{-2} h^{-1}), E_a is the activation energy associated with the permeation process (kJ mol^{-1}), R is the gas constant (kJ mol^{-1} K^{-1}), and T is absolute temperature (K). By taking the log of both sides of Eq. (8) and using R (8.314 × 10^{-3} kJ mol^{-1} K^{-1}), the E_a can be evaluated.

Supplementary Figures

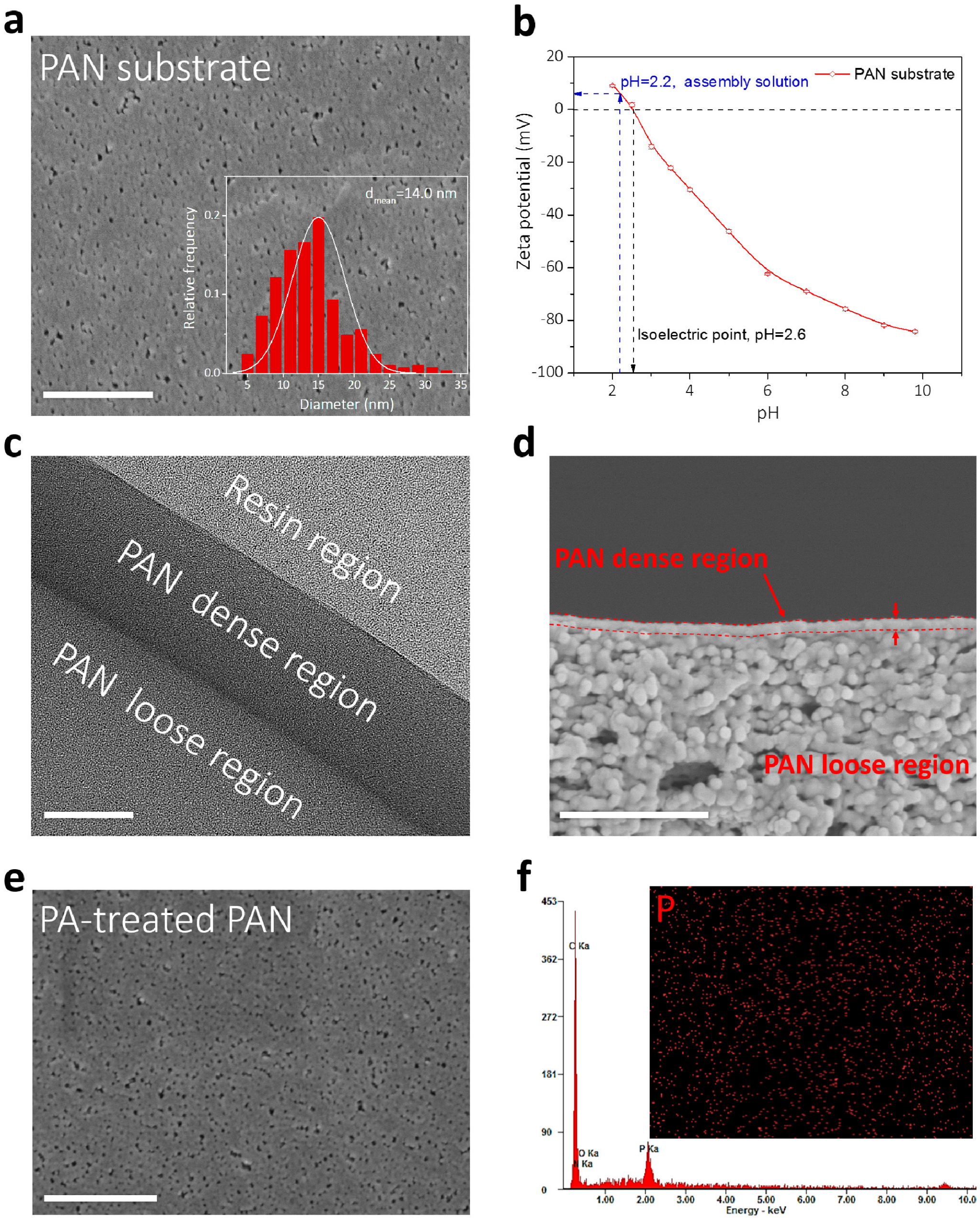

Supplementary Figure 1. (a) The surface morphology of PAN substrate. Scale bar: 500 nm. Inset: Pore size distribution of PAN substrate, estimated by an image analysis software titled Nano Measure. (b) The zeta potential of PAN substrate under varied pH condition. (c) The cross-section TEM image of PAN substrate. Scale bar: 20 nm. (d) The cross-section SEM image of PAN substrate. Scale bar: 500 nm (e) SEM image of PA-treated PAN substrate. (f) EDS mapping result of PA-treated PAN membrane. Inset: Distribution of phosphorus on PA-treated PAN substrate. Error bars represent standard deviations for 3 measurements.

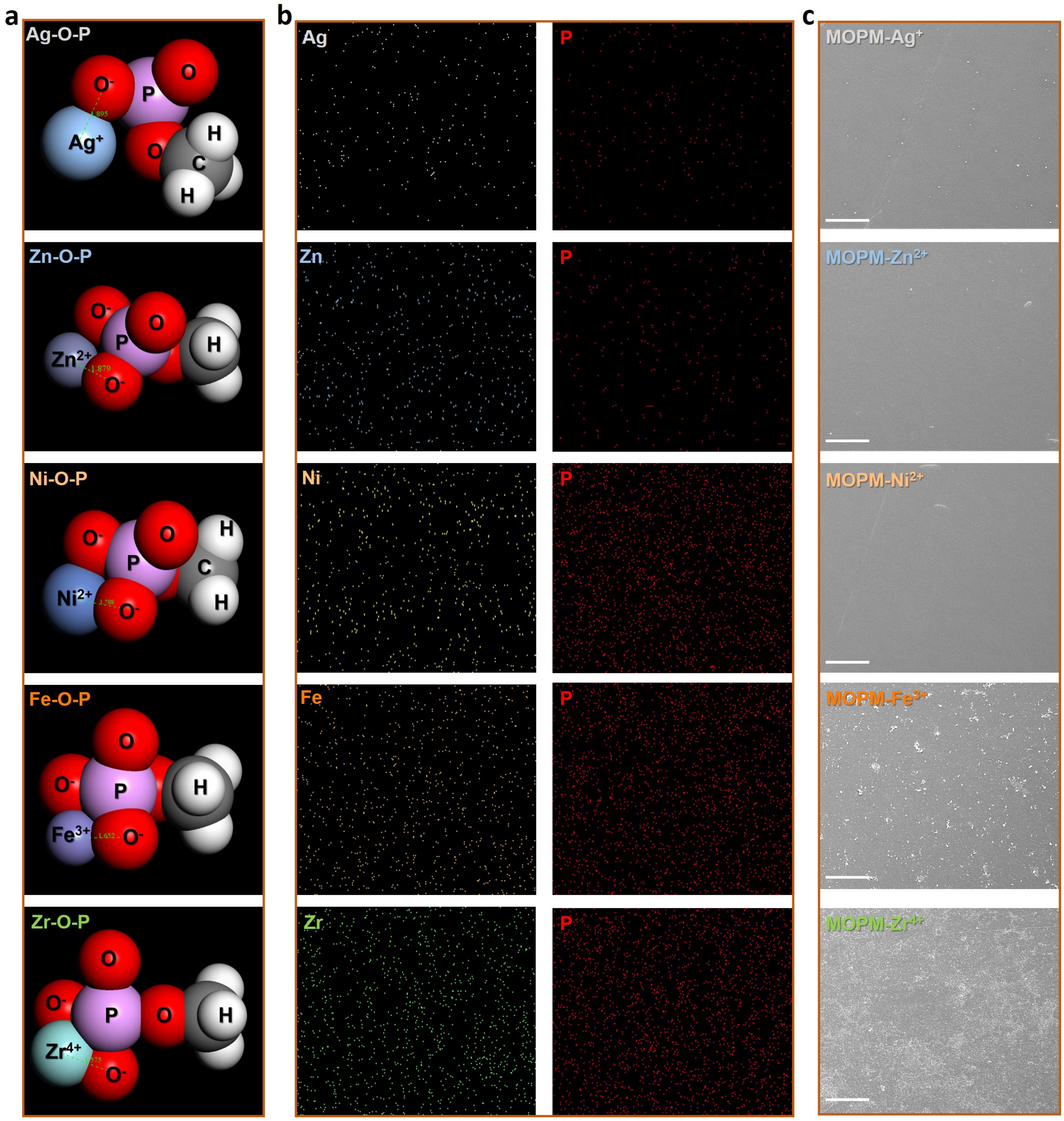

Supplementary Figure 2. (a) Simulated coordination mode between metal ion and methyl phosphate. (b) EDX mapping and (c) related SEM images of MOPMs. Scale bar: 5 μm.

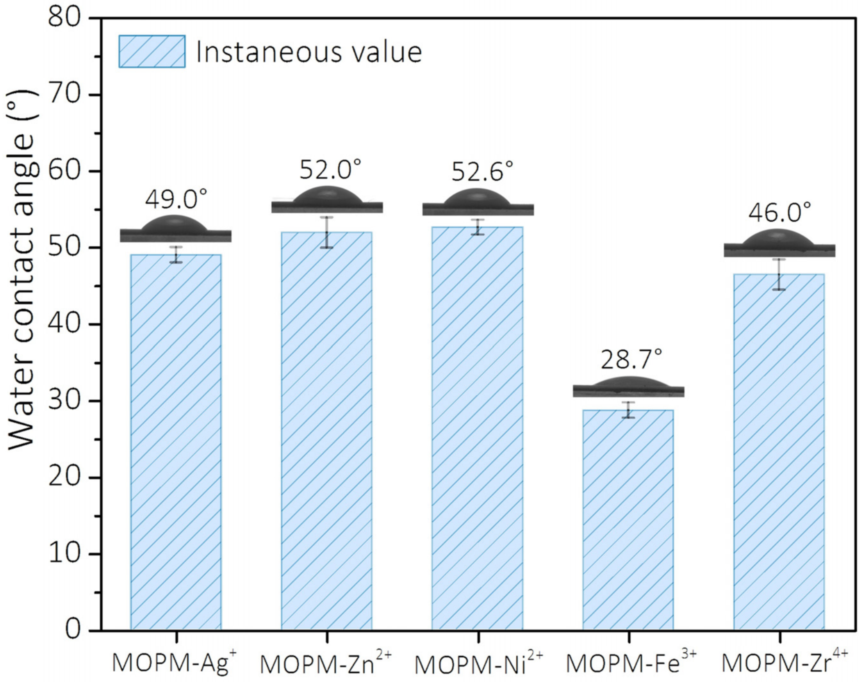

Supplementary Figure 3. Instantaneous water contact angles of MOPMs. Error bars represent standard deviations for 3 measurements.

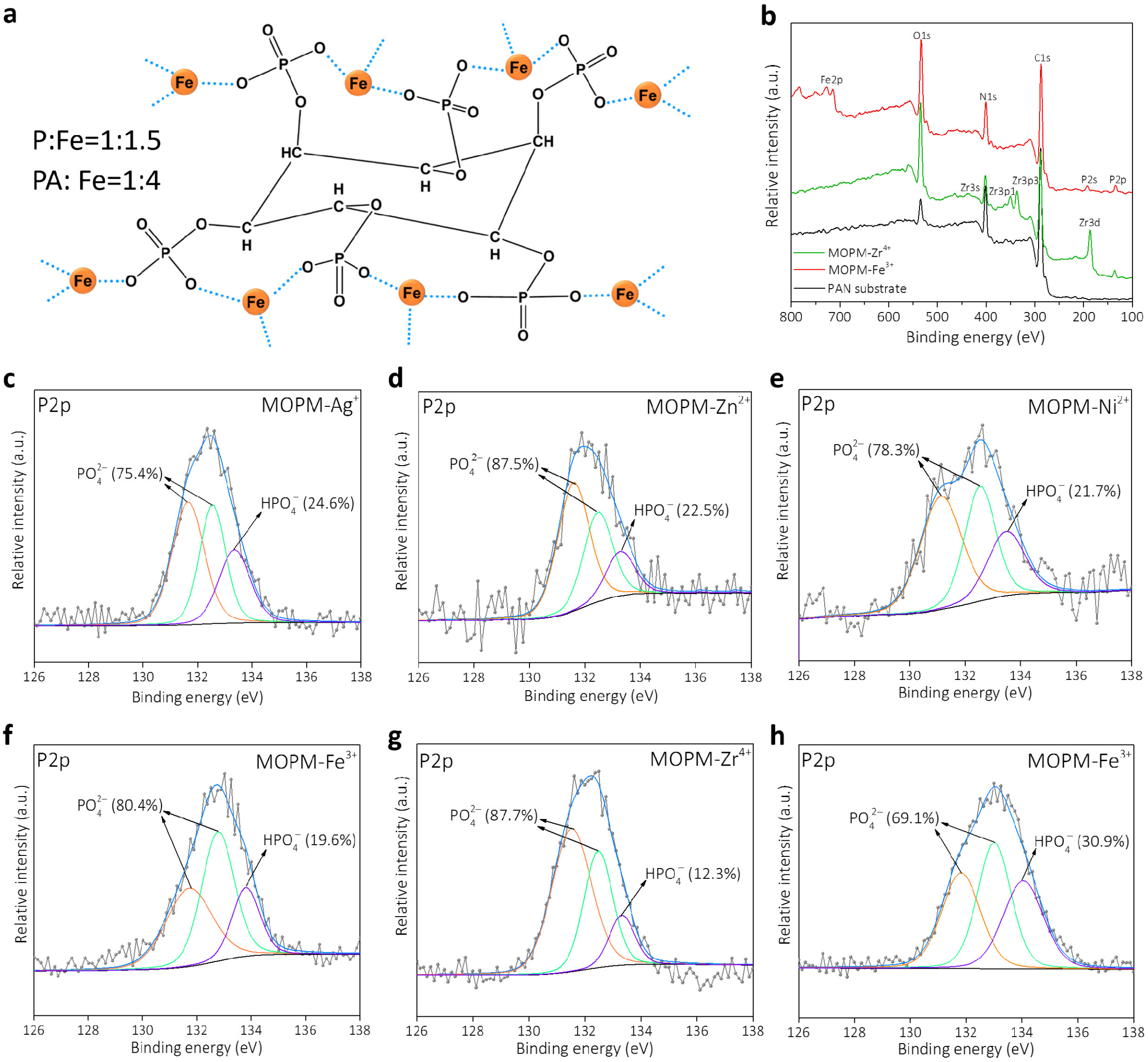

Supplementary Figure 4. (a) Illustration of idealized MOPM-Fe^{3+} with totally cross-linking structure. (b) The XPS results of PAN substrate and MOPM-Fe^{3+} and MOPM-Zr^{4+}. (c–h) High-resolution XPS of MOPMs. For (c-g) the PA/M ratio was 1:7 while for (h) the PA/Fe ratio was 1:0.5.

Note: The detected O element in PAN substrate demonstrated the partial hydrolization of C≡N groups into COOH groups. The greatly increased oxygen content was attributed to the abundant O ratio of phytic acid (44.4%). The detected N element in MOPM/PAN membranes was probably because the membrane thickness was less than the detection depth of XPS. The deconvolution of P2p revealed the coordination strength between phosphate group and metal ion in MOPMs. The deprotonation of phosphate group was the prerequisite of metal-organic coordination. Therefore, the stronger the metal-organic interaction was, the more PO_4^{2-} would be in MOPMs. According to the literature[5], the fitted peaks at 132.1 eV and 132.8 eV were attributed to deprotonated PO_4^{2-} and another fitted peak at 133.8 eV corresponded to HPO_4^{-}.

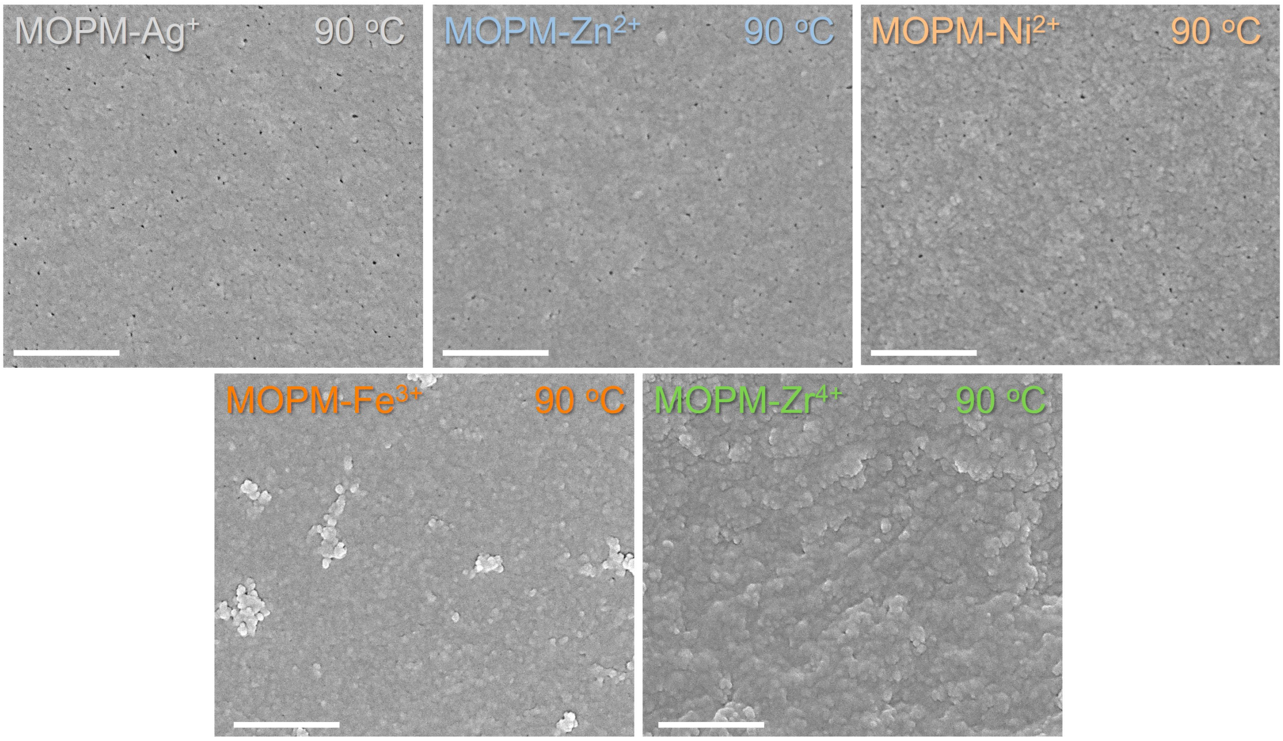

Supplementary Figure 5. SEM images of MOPMs prepared under high curing temperatures (90 °C). Scale bar: 500 nm.

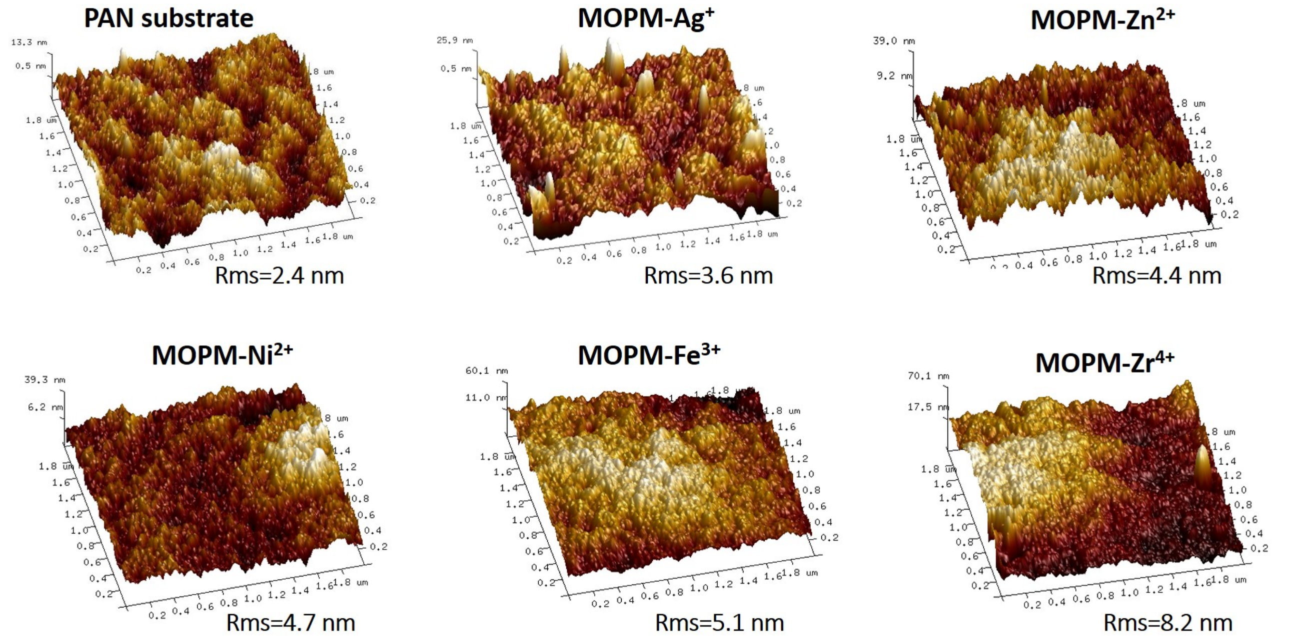

Supplementary Figure 6. The AFM images of PAN substrate and MOPMS. AFM images were exported by bundled software (NanoScopeAnalysis Version1.9, Bruker Dimension Icon).

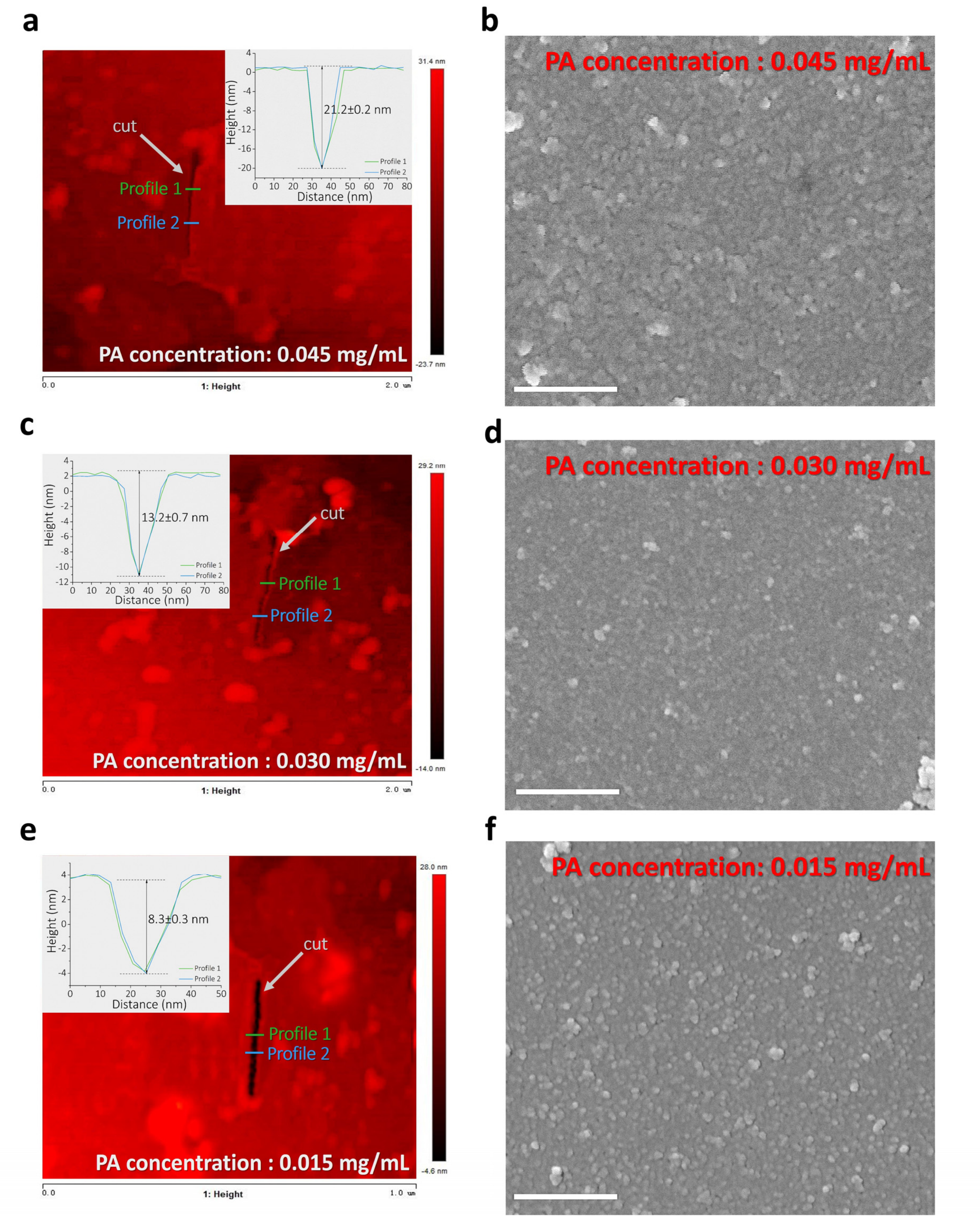

Supplementary Figure 7. (a), (c) and (e), AFM images of the transferred MOPM-Fe^{3+} with PA concentration of 0.045 mg/mL, 0.030 mg/mL and 0.015 mg/mL, respectively. The membranes were scratched with a sharp AFM probe with approximately 2 nN applied force for thickness detection. Insets: height profiles near the scratch. AFM images were exported by bundled software (NanoScopeAnalysis Version1.9, Bruker Dimension Icon). (b), (d) and (f), Surface morphologies of MOPM-Fe^{3+} with different PA concentration in assembly solution. The PA/Fe mole ratio and assembly time were fixed at 1:7 and 60 min, respectively. Scale bar: 500 nm.

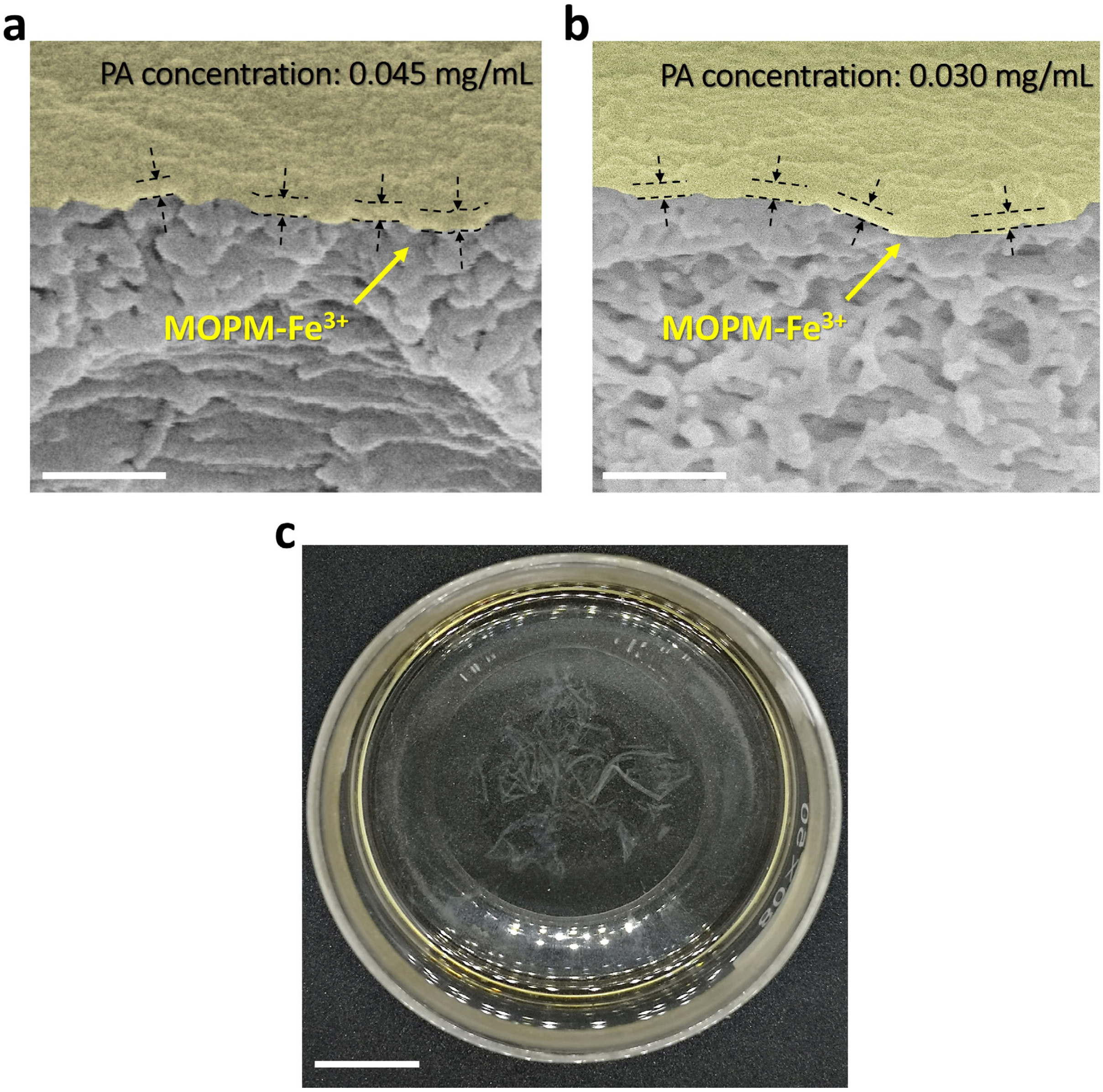

Supplementary Figure 8. (a) and (b), Cross-section images of the MOPM-Fe^{3+} on PAN substrate with PA concentration of 0.045 mg/mL and 0.030 mg/mL, respectively. Scale bar: 100 nm. False color was used to singularize the MOPM-Fe^{3+}. (c) Substrate-free MOPM-Fe^{3+} in DMF solution. The PA concentration, PA/Fe ratio and assembly time were 0.015 mg/mL, 1:7 and 60 min, respectively. Scale bar: 2 cm.

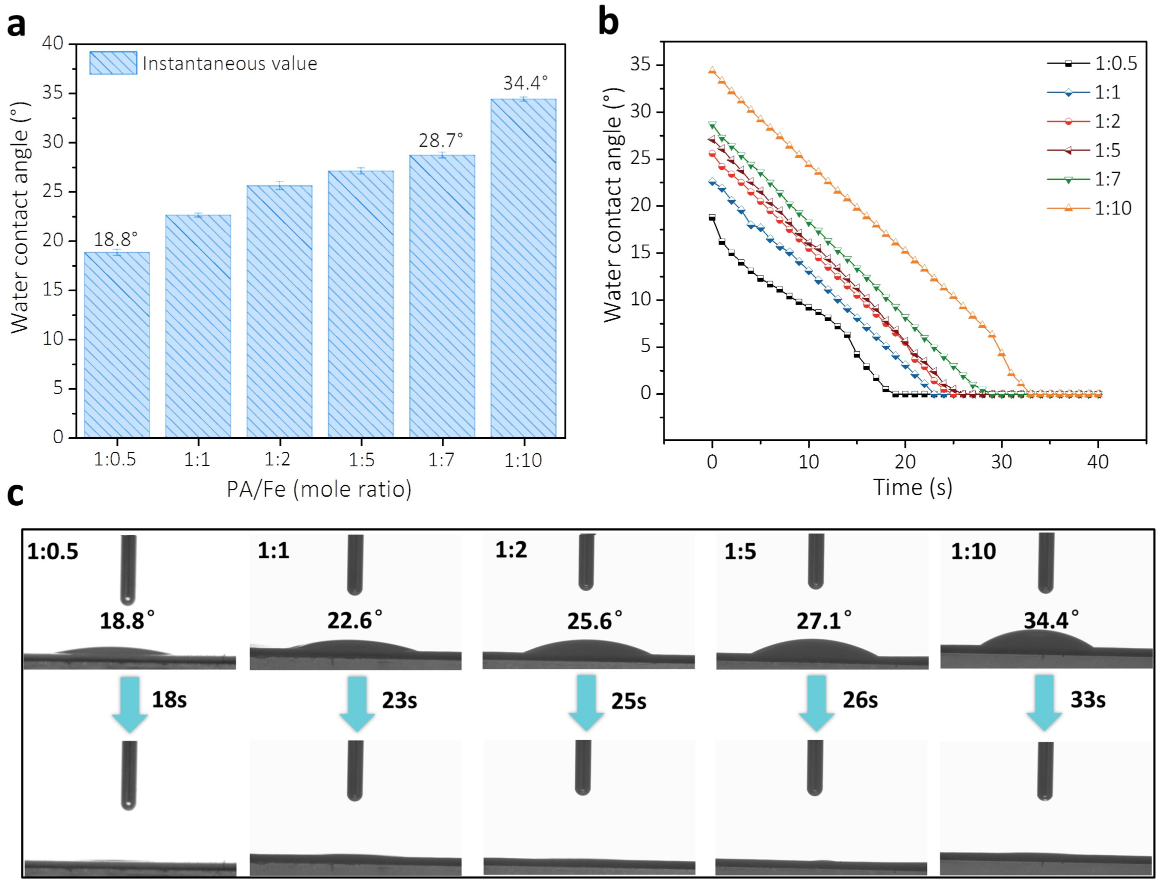

Supplementary Figure 9. (a) Water contact angles of MOPM-Fe^{3+} with varied PA/Fe ratio. (b) Dynamic water contact angles of MOPM-Fe^{3+} with varied PA/Fe ratio. (c) Digital photo images of water droplets on MOPM-Fe^{3+} with varied PA/Fe ratio.

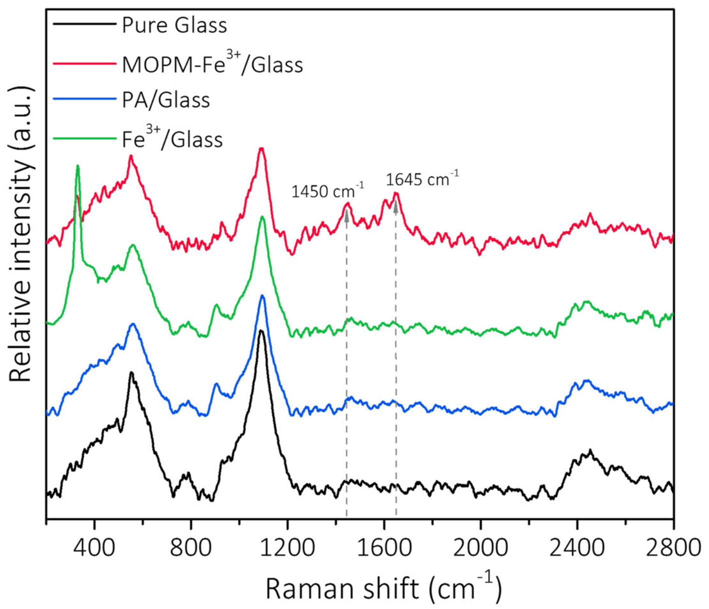

Supplementary Figure 10. Raman spectra of Fe^{3+}, PA and MOPM-Fe^{3+} on glass substrate.

Note: The emerging Raman shift at 1450 and 1645 cm^{-1} verified the coordination interaction between phosphate and Fe^{3+}.

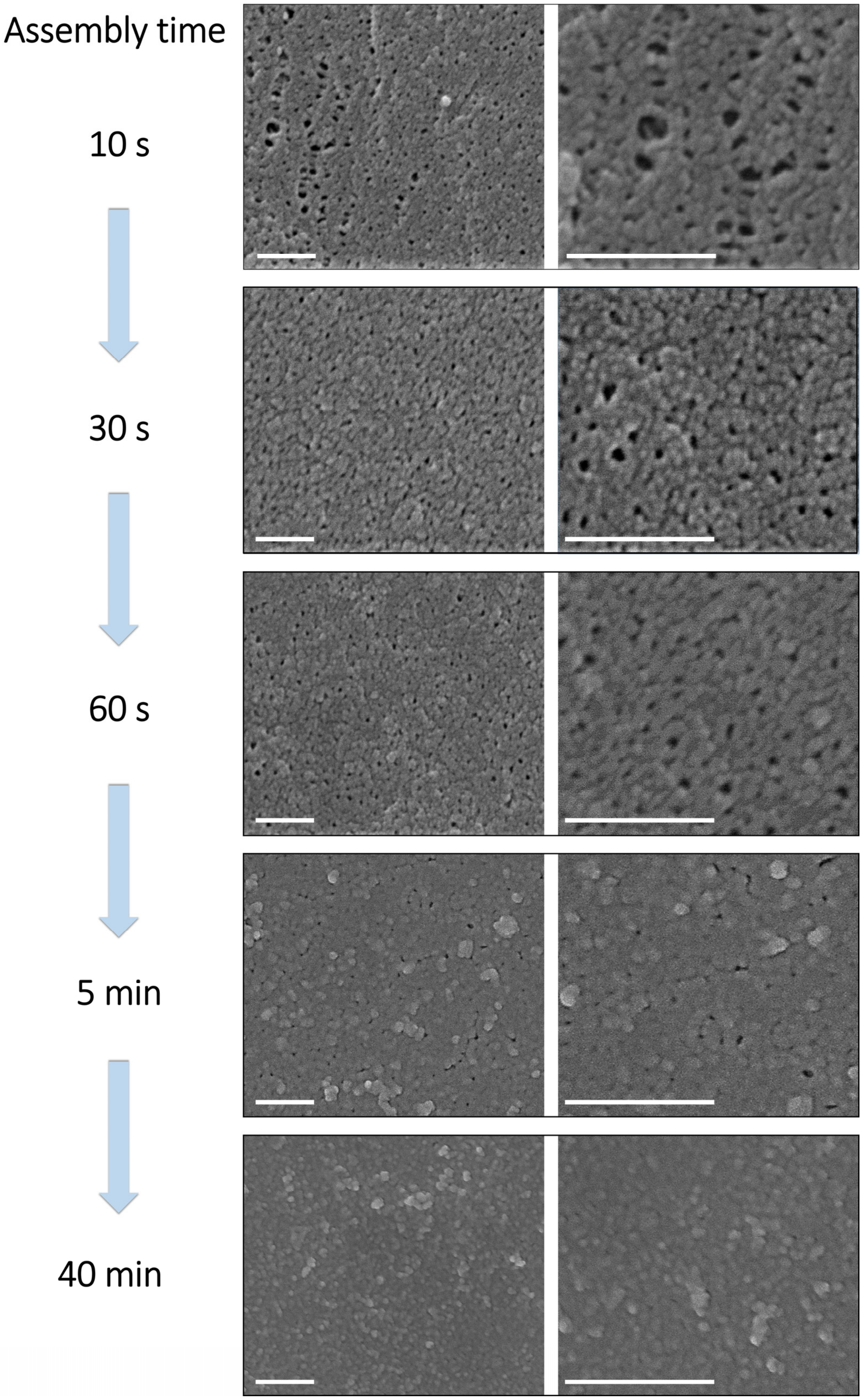

Supplementary Figure 11. The growing process of MOPM-Fe^{3+} on PAN substrate. The PA concentration and PA/Fe ratio were 0.015 mg/mL and 1:7, respectively. Scale bar: 200 nm.

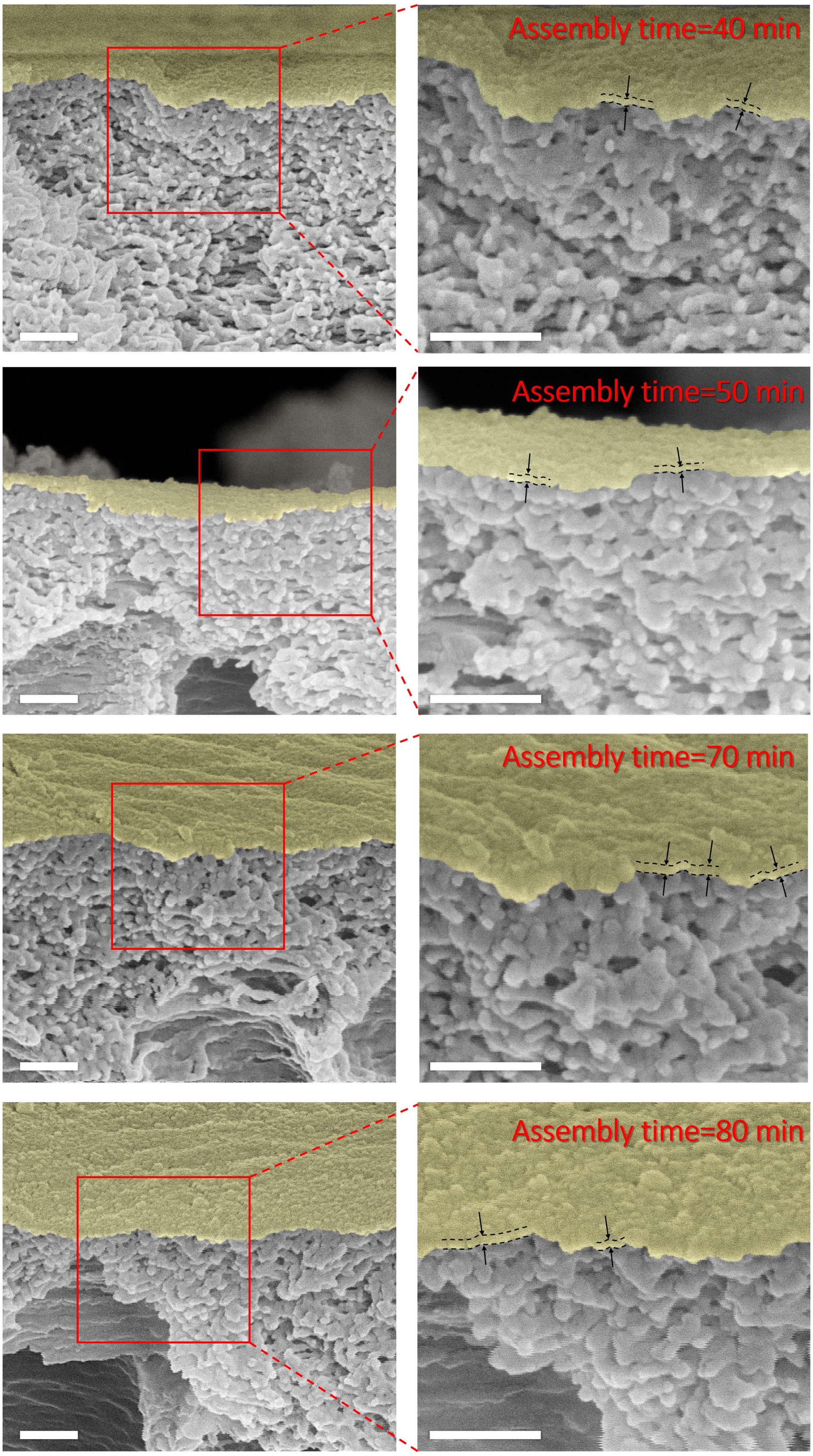

Supplementary Figure 12. The cross-section images of MOPM-Fe^{3+} with varied assembly time. The PA concentration and PA/Fe ratio were 0.015 mg/mL and 1:7, respectively. Scale bar: 100 nm.

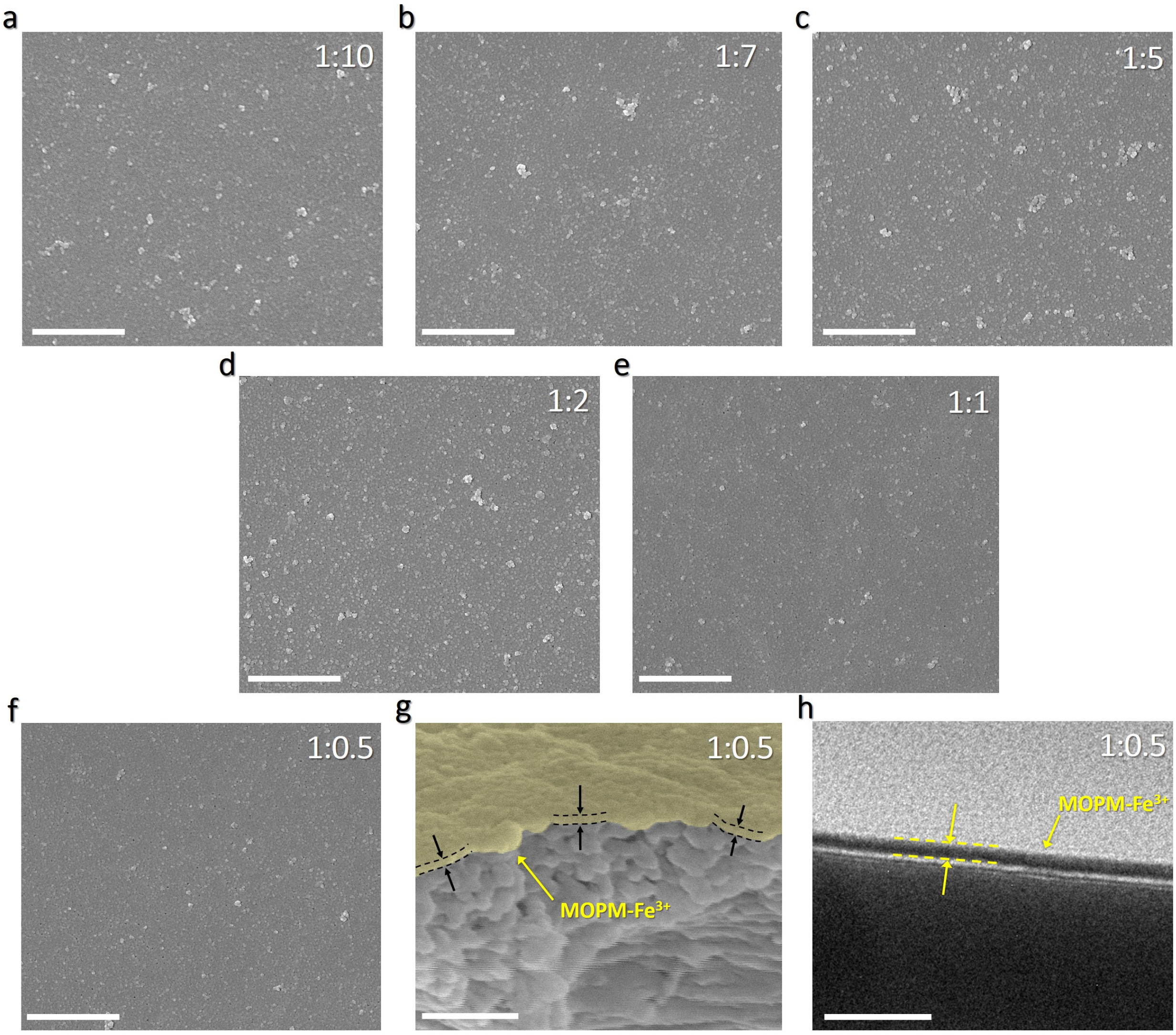

Supplementary Figure 13. (a-f) Surface morphologies of MOPM-Fe^{3+} with different PA/Fe ratio. Scale bar: 500 nm. (g) Cross-section morphology of MOPM-Fe^{3+} with PA/Fe ratio of 1:0.5. Scale bar: 100 nm. (h) TEM image of MOPM-Fe^{3+} with PA/Fe ratio of 1:0.5 on PAN substrate. Scale bar: 50 nm. The PA concentration and assembly time were fixed at 0.015 mg/mL and 60 min, respectively.

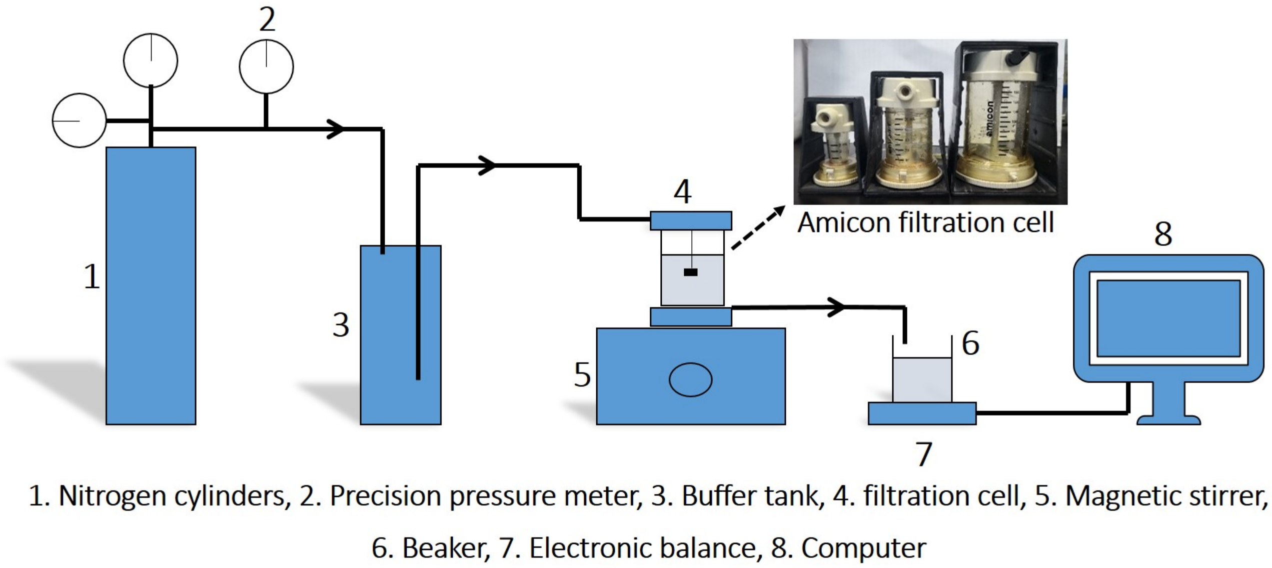

Supplementary Figure 14. The schematic illustration of filtration system. The values measured with electronic balance were collected by computer.

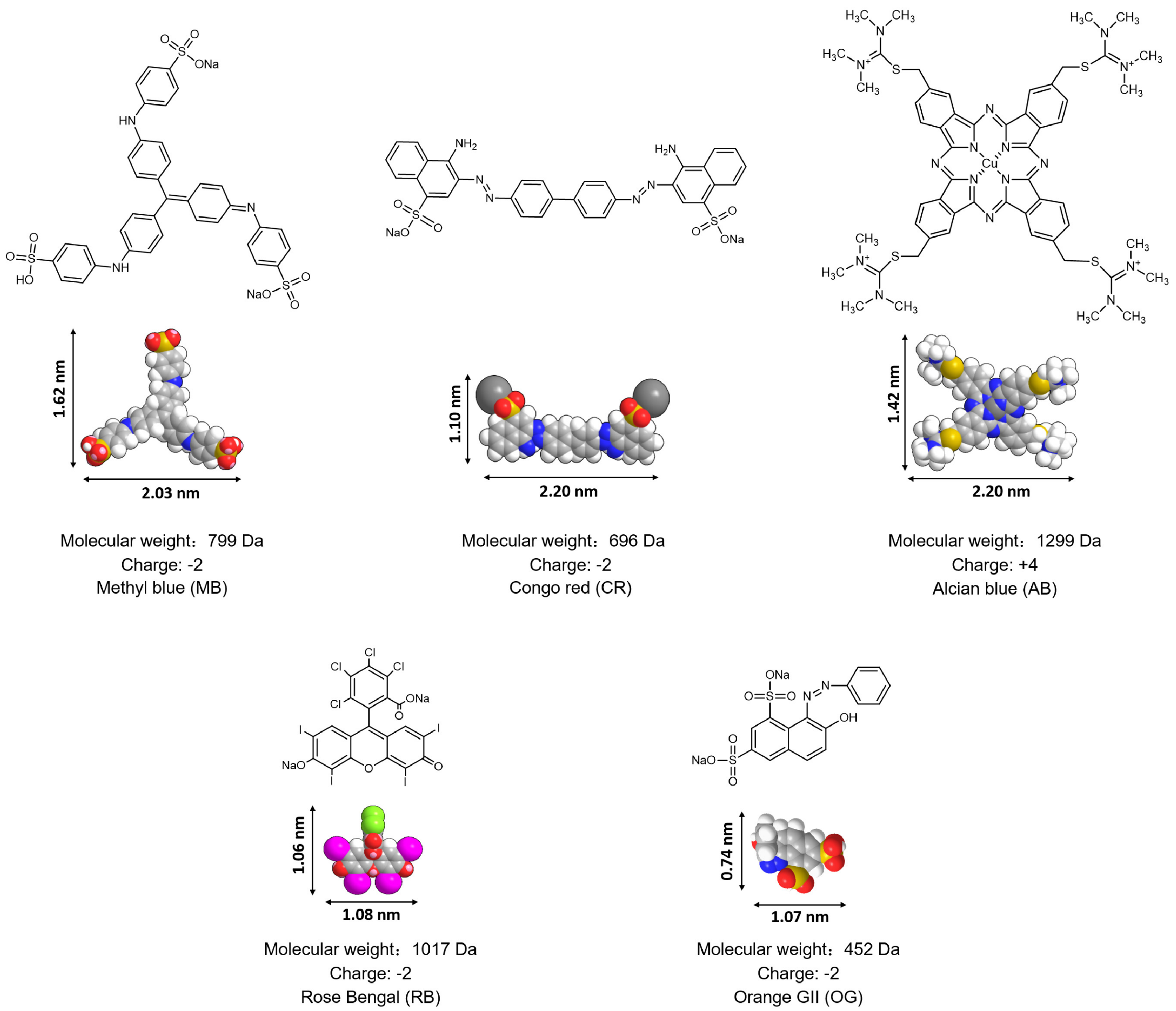

Supplementary Figure 15. Structural information of organic dyes. The molecular configuration was calculated by Chem3D software using Molecular Mechanism 2 method[2].

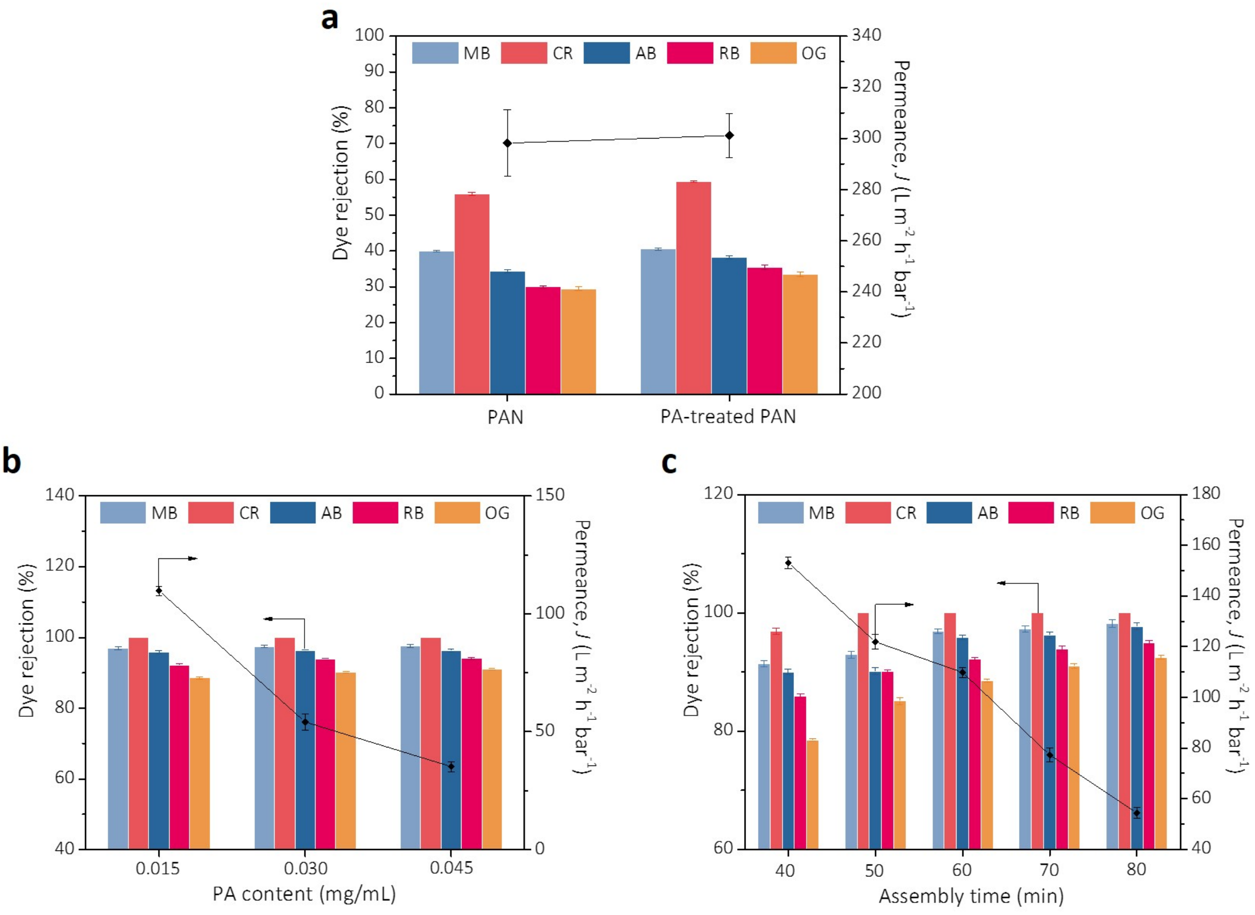

Supplementary Figure 16. (a) Filtration performance of PAN and PA-treated PAN. (b) and (c) Filtration performance of MOPM-Fe^{3+}/PAN membranes with varied PA concentration and assembly time, respectively. The dye concentration was 100 ppm. The PA concentration, PA/Fe ratio and assembly time were fixed at 0.015 mg/mL, 1:7 and 60 min, respectively. Error bars represent standard deviations for 3 measurements.

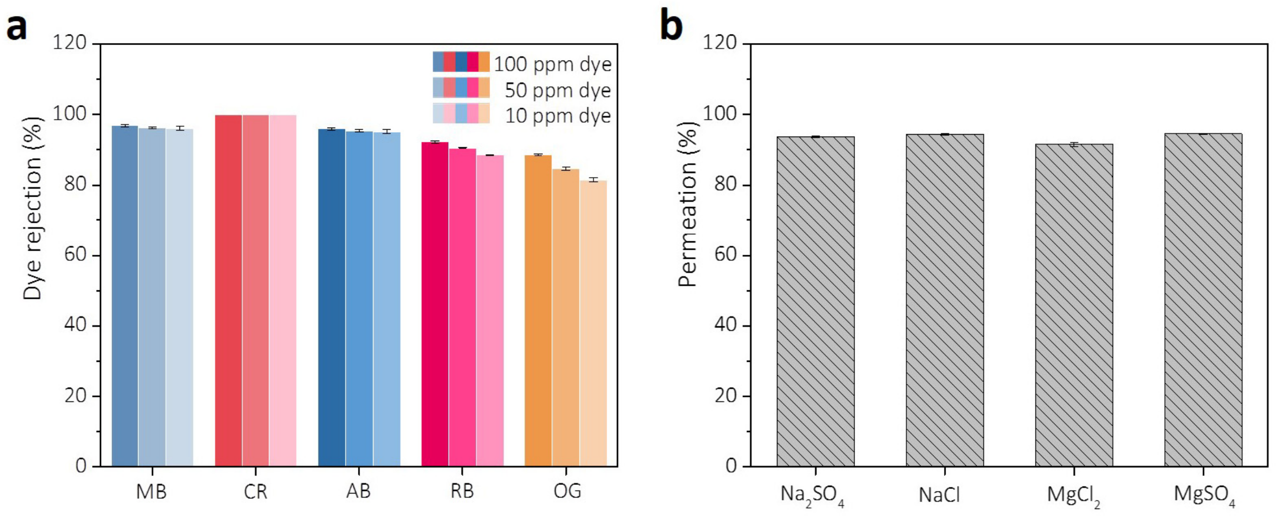

Supplementary Figure 17. (a) Dye rejections of MOPM-Fe^{3+}/PAN membrane with different feed concentration (100 ppm, 50 ppm and 10 ppm). (b) Permeation of different salts for MOPM-Fe^{3+}/PAN membrane. The salt concentration was 1000 ppm. The PA concentration, PA/Fe ratio and assembly time were fixed at 0.015 mg/mL, 1:7 and 60 min, respectively. Error bars represent standard deviations for 3 measurements.

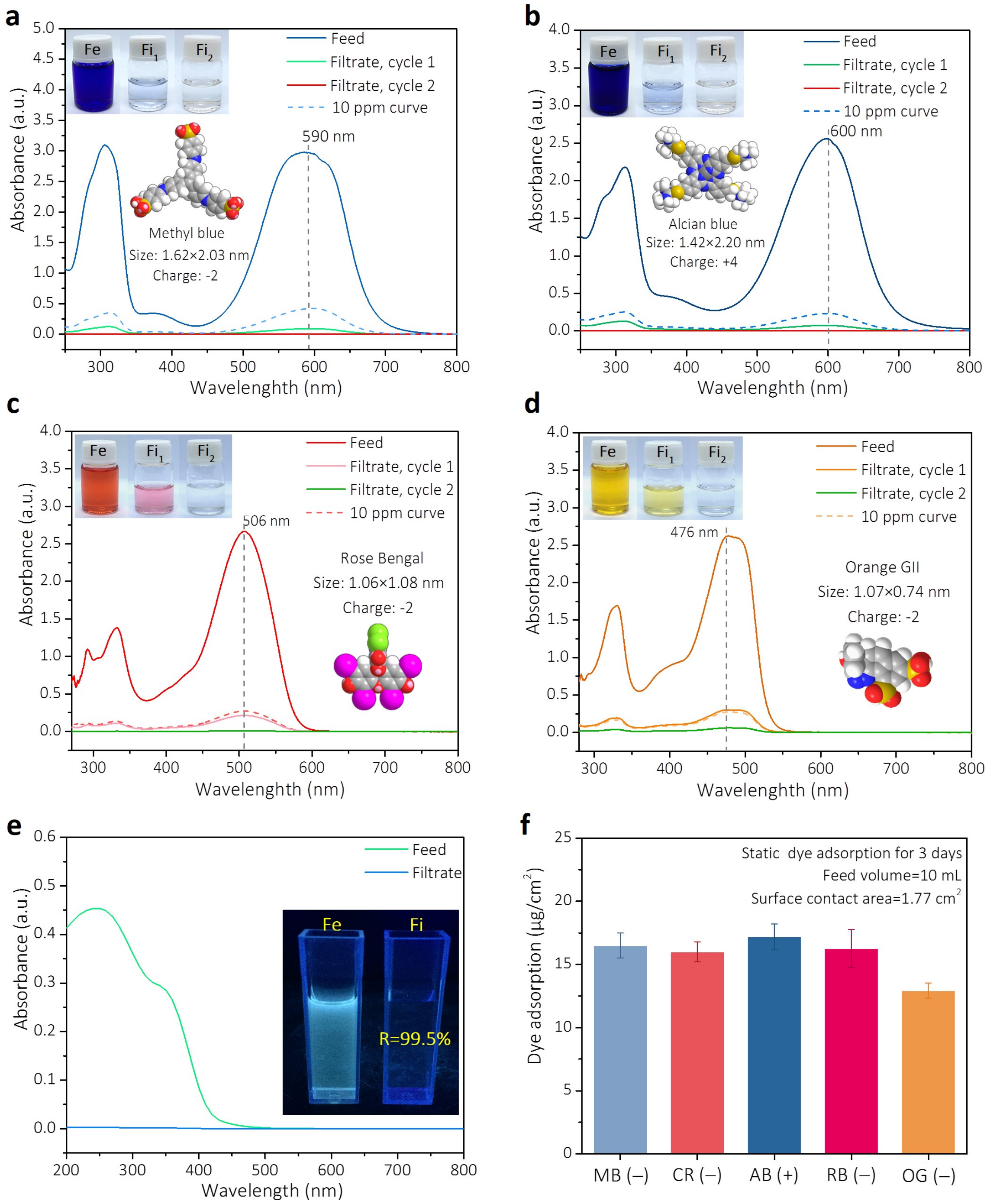

Supplementary Figure 18. Ultraviolet-visible absorption spectra of (a) Methyl blue (MB), (b) Alcian blue (AB), (c) Rose Bengal (RB), (d) Orange GII (OG) and (e) GQDs in feed and filtrate of MOPM-Fe^{3+}/PAN membrane. Inset: Digital photo images of feed (Fe) and filtrate (Fi) (top left) and molecular structure of MB and AB. The filtrate of cycle 1 (5 mL feed) was used as the feed of cycle 2. (f) The dye adsorption of MOPM-Fe^{3+}/PAN membrane. The PA concentration, PA/Fe ratio and assembly time were fixed at 0.015 mg/mL, 1:7 and 60 min, respectively. Error bars represent standard deviations for 3 measurements.

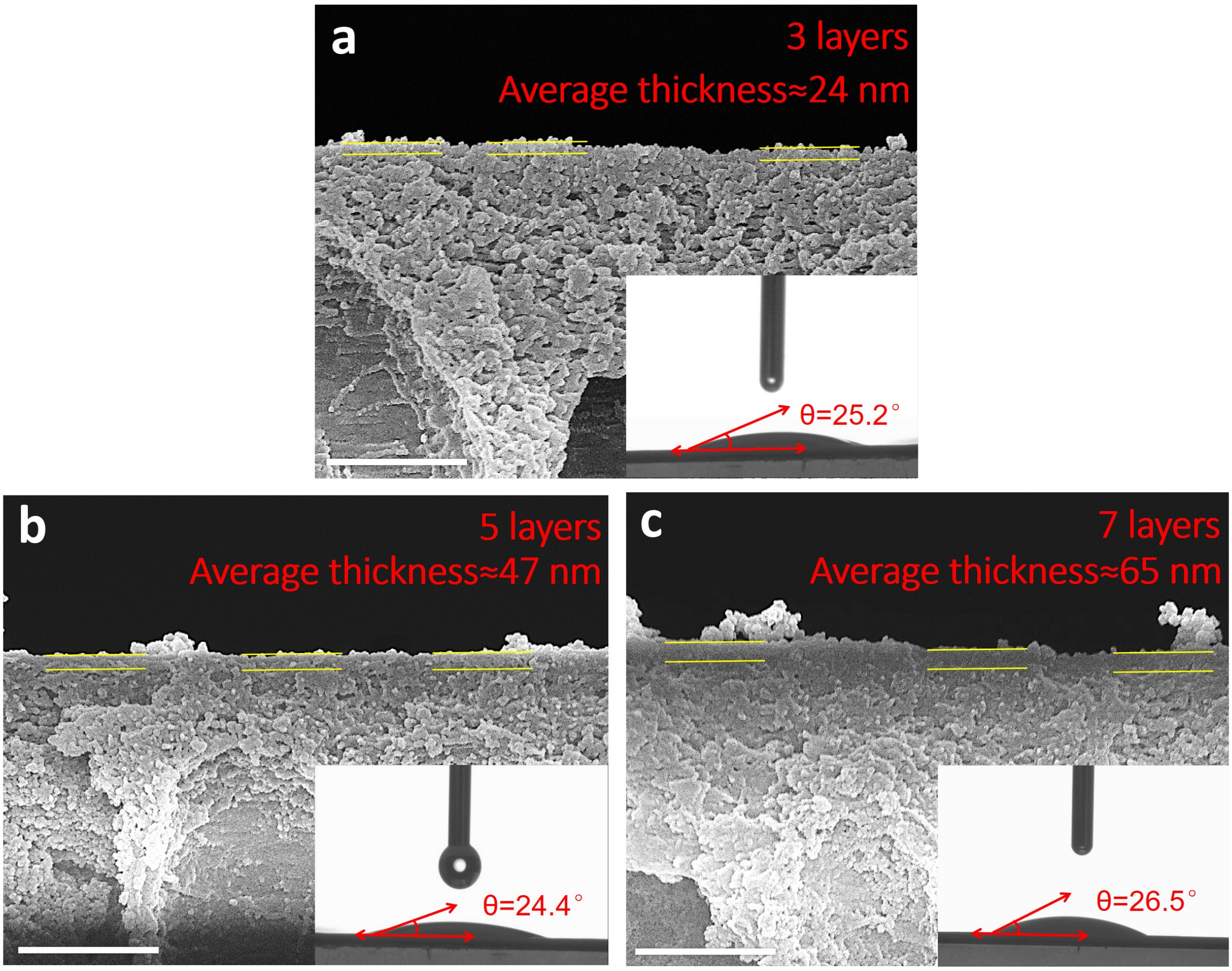

Supplementary Figure 19. SEM images of cross-section morphology of MOPM-Fe^{3+}/PAN membrane with (a) 3, (b) 5 and (c) 7 assembly layers. Scale bar: 500 nm. Inset: water contact angles of membranes.

Note: The layer-by-layer assembly of MOPM-Fe^{3+} on PAN substrate was conduct to obtain MOPM-Fe^{3+}/PAN membranes with different skin layer thicknesses. The PA concentration, PA/Fe ratio and assembly time were fixed at 0.015 mg/mL, 1:7 and 60 min, respectively. The procedure for a single MOPM-Fe^{3+} layer was defined as a cycle. The next cycle was carried out on the surface of obtained MOPM-Fe^{3+} layer in last cycle.

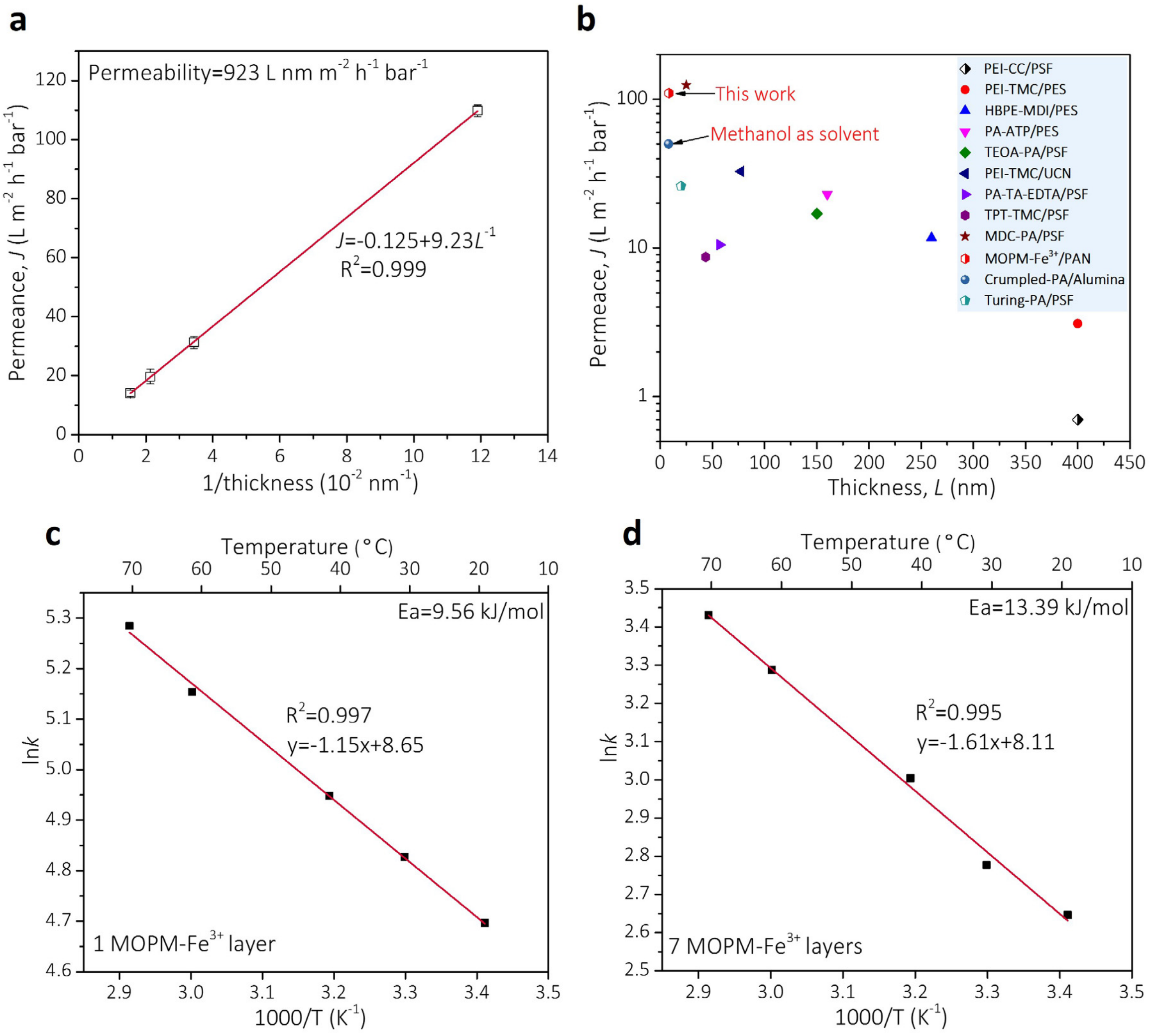

Supplementary Figure 20. (a) Permeance as a function of inverse thickness for MOPM-Fe^{3+}/PAN membranes with different skin layer thicknesses. (b) Summary of thicknesses and water permeances of polymeric membranes in literatures. (c) and (d) Arrhenius plot of the water permeation rate (ln*k*) versus inverse temperature (1000/T) for MOPM-Fe^{3+}/PAN membranes with 1 and 7 MOPM-Fe^{3+} layers, respectively. The PA concentration, PA/Fe ratio and assembly time were 0.015 mg/mL, 1:7 and 60 min, respectively. Error bars represent standard deviations for 3 measurements.

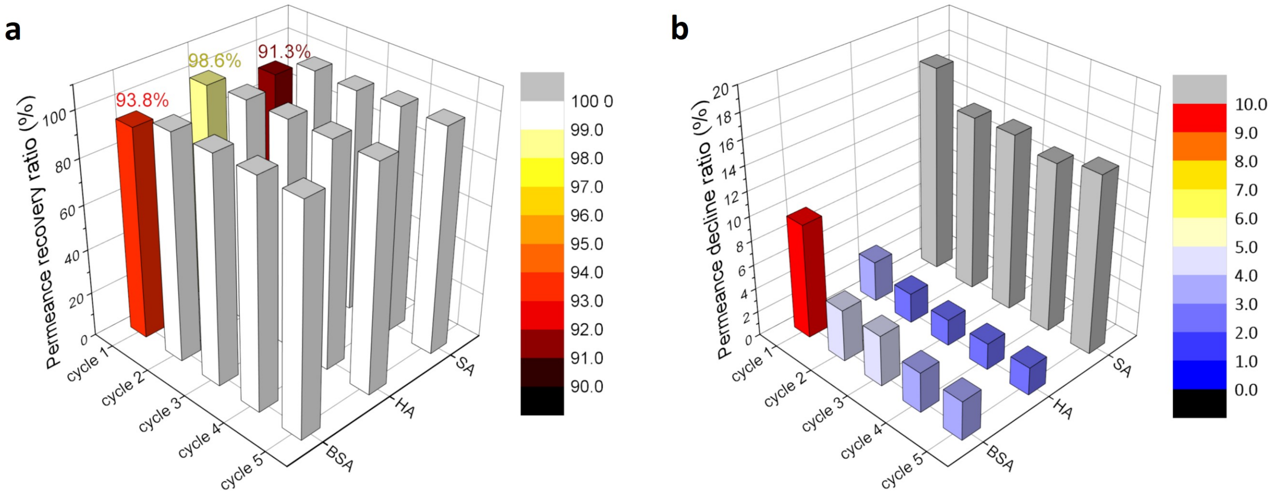

Supplementary Figure 21. (a) Permeance recovery ratio and (b) permeance decline ratio of MOPM-Fe^{3+}/PAN membrane for each cycle in five-stage antifouling measurement. The PA concentration, PA/Fe ratio and assembly time were 0.015 mg/mL, 1:7 and 60 min, respectively.

Note: The total permeance decline ratio after five-stage antifouling measurement using BSA, HA and SA were 6.3%, 1.9% and 18.4%, respectively. The relatively high permeation decline with SA was probably because the higher viscosity of SA solution (~1.4 cP, M_w=48 kDa, 1000 ppm, 25 ºC) than DI water (~0.89 cP, 25 ºC)[6].

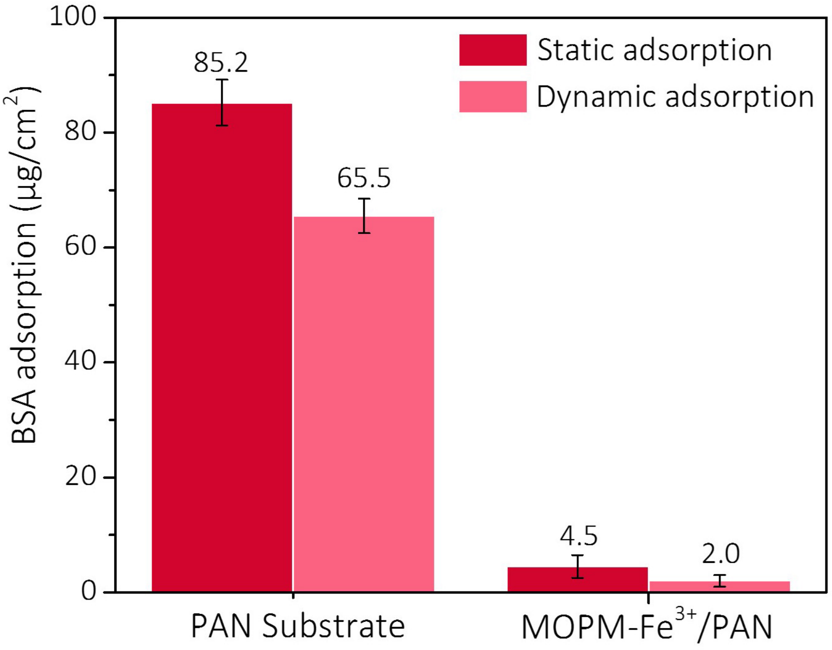

Supplementary Figure 22. BSA adsorption measurement of PAN and MOPM-Fe^{3+}/PAN membrane. The PA concentration, PA/Fe ratio and assembly time were 0.015 mg/mL, 1:7 and 60 min, respectively. Error bars represent standard deviations for 3 measurements.

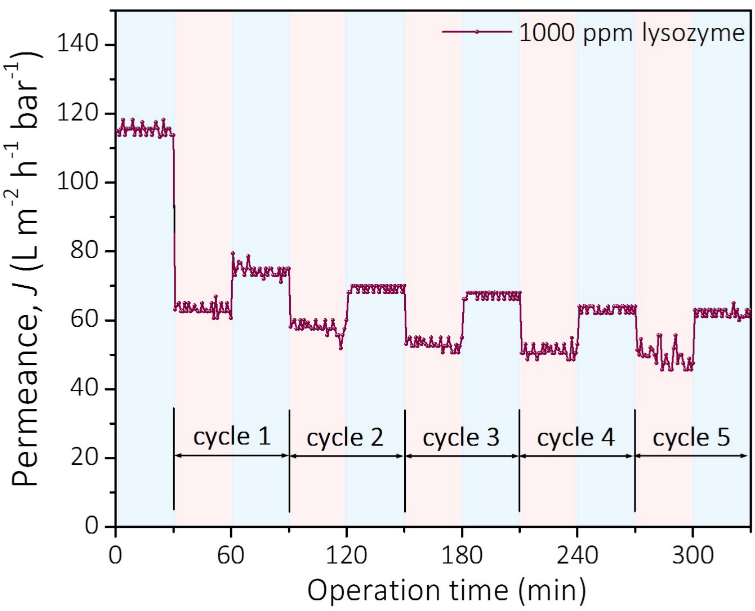

Supplementary Figure 23. Five-stage antifouling measurement of MOPM-Fe^{3+}/PAN membrane with 1000 ppm of lysozyme as feed at 1.0 bar. The PA concentration, PA/Fe ratio and assembly time were 0.015 mg/mL, 1:7 and 60 min, respectively.

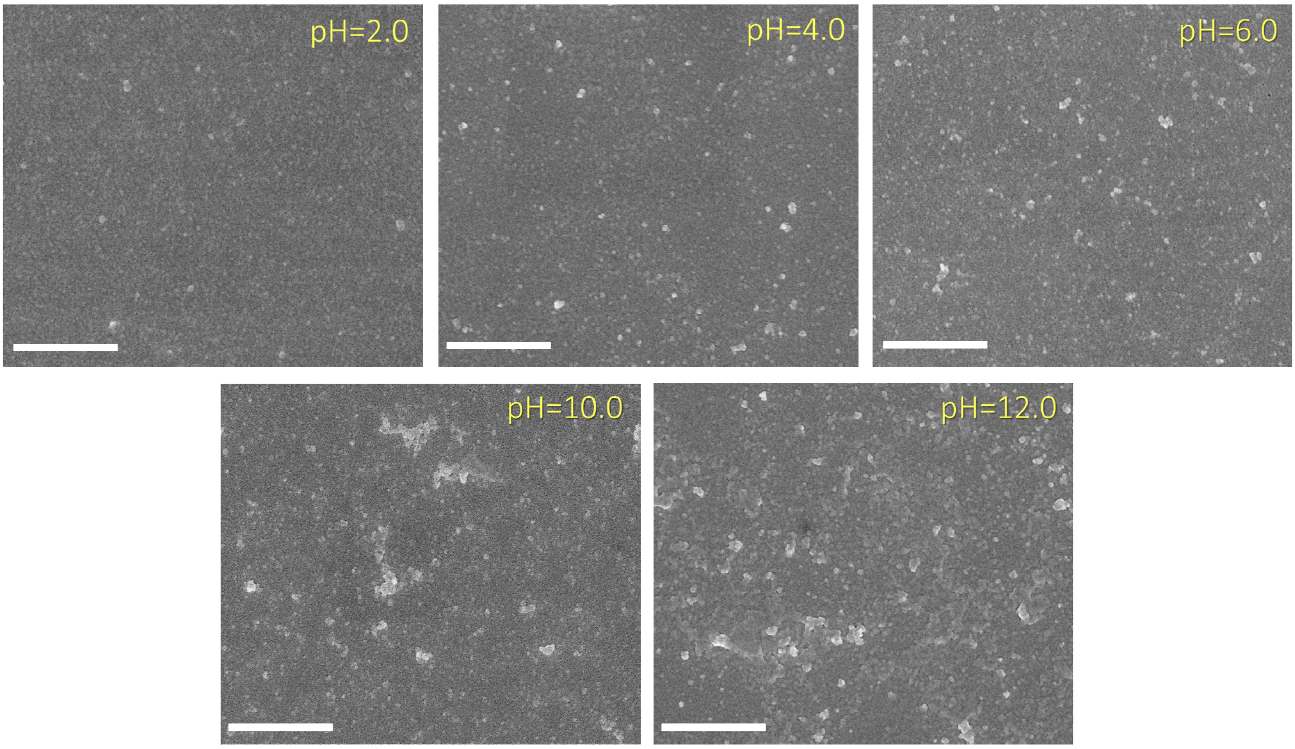

Supplementary Figure 24. SEM images of MOPM-Fe^{3+}/PAN membrane after immersed in varied pH solutions for 24 h. The PA concentration, PA/Fe ratio and assembly time were 0.015 mg/mL, 1:7 and 60 min, respectively. Scale bar: 500 nm.

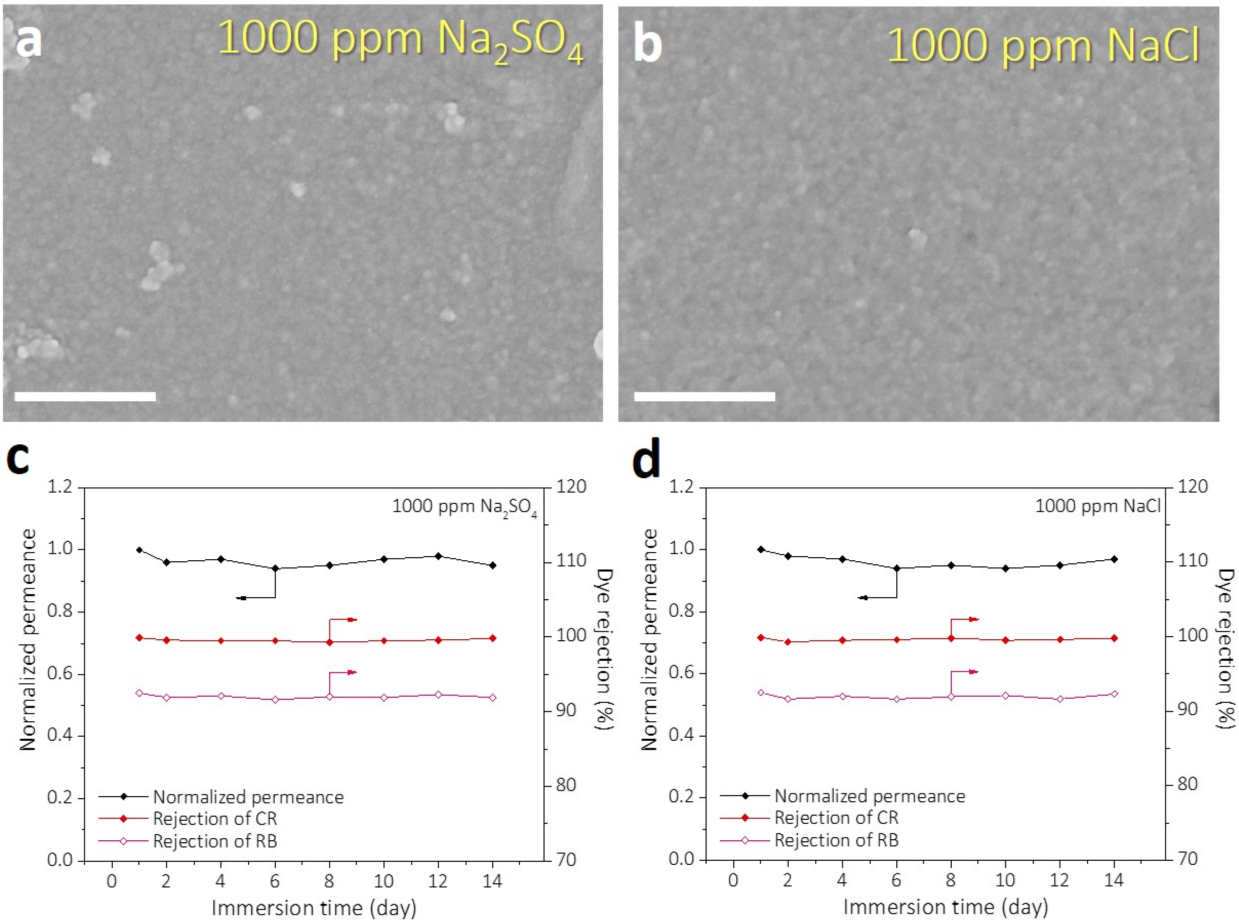

Supplementary Figure 25. (a) and (b) SEM images of MOPM-Fe^{3+}/PAN membrane after immersed in salt solution for 2 weeks. Scale bar: 500 nm. (c) and(d) Long-term stability of MOPM-Fe^{3+}/PAN membrane in Na_2SO_4 and NaCl solution.

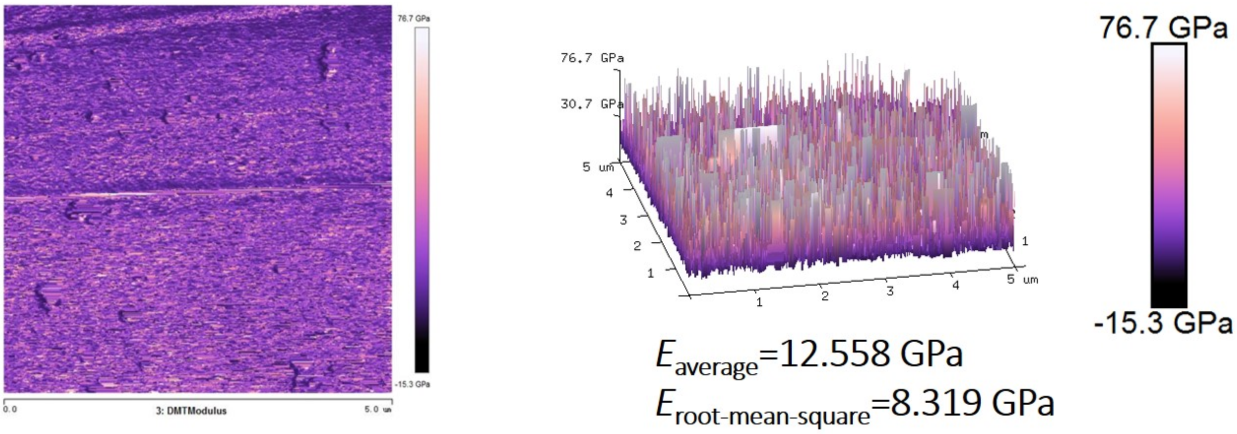

Supplementary Figure 26. Young's modulus (*E*) of MOPM-Fe^{3+}/PAN membrane. The PA concentration, PA/Fe ratio and assembly time were 0.015 mg/mL, 1:7 and 60 min, respectively. AFM images were exported by bundled software (NanoScopeAnalysis Version1.9, Bruker Dimension Icon).

Supplementary Tables

Supplementary Table 1. Characteristics of transition metal ions used in this work.

Ion type	Radius, r (nm)[7]	Charge number, n	Ionization potential, I_n (eV)[c]	Category of Lewis acid
Ag^{+}	0.126	1	7.58	Soft acid
Zn^{2+}	0.074	2	17.96	Borderline acid
Ni^{2+}	0.069	2	18.17	Borderline acid
Fe^{3+}	0.065[a], 0.055[b]	3	30.65	Hard acid
Zr^{4+}	0.072	4	34.34	Hard acid

[a] High spin.

[b] Low spin.

[c] Data obtained from *Atomic & Molecular Database* (http://www.camdb.ac.cn/nsdc/a_ip.asp) of Beijing Institute of Applied Physics and Computational Mathematics.

Supplementary Table 2. Simulation results of coordination between transition metal ions and methyl phosphate ester.

Metal ion type	$E_{(phosphate/metal\ ion)}$ (Hartree)	$E_{(methyl\ phosphate)}$ (Hartree)	$E_{(metal\ ion)}$ (Hartree)	Binding energy (Hartree)[a]	Binding energy (kJ mol^{-1})[a]	M−O bond length (Å)[b]
Ag^{+}	−867.18	−682.16	−184.94	−0.080	−210.0	1.895
Zn^{2+}	−936.91	−682.21	−254.614	−0.090	−236.3	1.879
Ni^{2+}	−876.00	−682.20	−193.7	−0.100	−262.5	1.788
Fe^{3+}	−825.45	−682.14	−143.19	−0.120	−315.1	1.652
Zr^{4+}	−753.49	−682.00	−71.33	−0.160	−420.1	1.575

[a] Conversion basis: 1 Hartree=2625.5 kJ/mol.

[b] “M-O” bond represent “Metal-oxygen” coordination bond.

Supplementary Table 3. Summary of elemental ratio of MOPMs in this work.

Membrane name	Metal salt type	Mole ratio[a] (PA:M)	Assembly time (min)	Mole ratio[b] (P:M)	Mole ratio[b] (PA:M)
MOPM-Ag^{+}	$AgNO_3$	1:7	60	5.51:1	1:1.1
MOPM-Zn^{2+}	$ZnCl_2$	1:7	60	4.13:1	1:1.5
MOPM-Ni^{2+}	$NiCl_2 \cdot 6H_2O$	1:7	60	4.40:1	1:1.4
MOPM-Fe^{3+}	$FeCl_3 \cdot 6H_2O$	1:7	60	1.81:1	1:3.3
MOPM-Zr^{4+}	$Zr(NO_3)_4 \cdot 5H_2O$	1:7	60	1.46:1	1:4.1
**Condition experiment of MOPM-Fe^{3+}**					
MOPM-Fe^{3+}-1	$FeCl_3 \cdot 6H_2O$	1:0.5	60	2.69:1	1:2.2
MOPM-Fe^{3+}-2	$FeCl_3 \cdot 6H_2O$	1:1	60	2.39:1	1:2.5
MOPM-Fe^{3+}-3	$FeCl_3 \cdot 6H_2O$	1:2	60	2.02:1	1:3.0
MOPM-Fe^{3+}-4	$FeCl_3 \cdot 6H_2O$	1:5	60	1.87:1	1:3.2
MOPM-Fe^{3+}-5	$FeCl_3 \cdot 6H_2O$	1:7	60	1.81:1	1:3.3
MOPM-Fe^{3+}-6	$FeCl_3 \cdot 6H_2O$	1:7	60	1.79:1	1:3.4
MOPM-Fe^{3+}-7	$FeCl_3 \cdot 6H_2O$	1:7	60	1.72:1	1:3.5
MOPM-Fe^{3+}-8	$FeCl_3 \cdot 6H_2O$	1:10	60	1.59:1	1:3.8
MOPM-Fe^{3+}-9	$FeCl_3 \cdot 6H_2O$	1:7	40	1.92:1	1:3.1
MOPM-Fe^{3+}-10	$FeCl_3 \cdot 6H_2O$	1:7	50	1.88:1	1:3.2
MOPM-Fe^{3+}-11	$FeCl_3 \cdot 6H_2O$	1:7	70	1.69:1	1:3.6
MOPM-Fe^{3+}-12	$FeCl_3 \cdot 6H_2O$	1:7	80	1.61:1	1:3.7

[a] The PA:M mole ratio in assembly solution.

[b] The mole ratio of MOPMs detected by EDX, where M represented Ag, Zn, Ni, Fe and Zr element.

Supplementary Table 4. Summary of thickness and water permeance of polymeric membranes in literatures.

Membrane name	Membrane thickness (nm)	Permeance ($L\ m^{-2}\ h^{-1}\ bar^{-1}$)	Ref.
PEI-CC/PSF	400	0.7	8
PEI-TMC/PES	400	3.1	9
HPBE-MDI/PES	260	11.7	10
PA-ATP/PES	160	23.0	11
TEOA-PA/PSF	150	17.0	12
PEI-TMC/UCN	77.4	32.7	13
PA-TA-DETA/PSF	56.9	10.5	14
TPT-TMC/PSF	43.6	8.68	15
MDC-PA/PSF	25	124.6	16
Turing-PA/PSF	20	26.0	17
MOPM-Fe^{3+}/PAN	8.3	109.8	This work
Controlled-IP/Alumina	~8	50 (methanol)	18

Supplementary Table 5. Comparison of the separation performance of advanced polymeric membranes in the literatures (in an order of permeance).

Membrane name	Permeance ($L\ m^{-2}\ h^{-1}\ bar^{-1}$)	Dye rejection	Dye feed concentration (ppm)	Ref.
MOPM-Fe^{3+}-1/PAN	160.3	94.1%, Congo red	100	this work
MOPM-Fe^{3+}-2/PAN	158	97.5%, Congo red	100	this work
MOPM-Fe^{3+}-9/PAN	153.1	91.4%, Methyl blue<br>99.9%, Congo red	100	this work
MOPM-Fe^{3+}-3/PAN	149.8	90.8%, Methyl blue<br>99.9%, Congo red<br>90.0%, Alcian blue	100	this work
MOPM-Fe^{3+}-10/PAN	121.9	92.8%, Methyl blue<br>100.0%, Congo red<br>90.1%, Alcian blue	100	this work
MOPM-Fe^{3+}-4/PAN	120.9	92.9%, Methyl blue<br>100.0%, Congo red<br>90.5%, Alcian blue	100	this work
MOPM-Fe^{3+}-5/PAN	109.8	96.9%, Methyl blue<br>100.0%, Congo red<br>95.9%, Alcian blue	100	this work
MOPM-Fe^{3+}-5/PAN	109.8	96.2%, Methyl blue<br>100.0%, Congo red<br>95.4%, Alcian blue	50	this work
MOPM-Fe^{3+}-5/PAN	109.8	96.1%, Methyl blue<br>100.0%, Congo red<br>95.2%, Alcian blue	10	this work

ZIF-8-PEI/HPAN	75.1	99.2%, Congo red<br>98.9%, Methyl blue	100	19
TA-Ni^{2+}/PES	56.1	95.3%, Rose Bengal<br>93.2%, Methyl blue	50	20
F-PDA/PES	46.1	99.0%, Congo red	100	21
TA-Fe^{III}/PES	27.8	94.8%, Orange GII<br>99%, Congo red	100	22
ZIF-8-PSS/HPAN	26.5	98.6%, Methyl blue	100	23
Ra-PDA-PEI/HPAN	26.2	98.1%, Reactive orange 16<br>>95%, Direct 23<br>>95%, Reactive blue 2<br>>80%, Rhodanile blue	500	24
PEI-GA/PAN	25.5	97.1%, Congo red	100	25
SiO_2-PSS-PES	23.3	>90%, reactive black 5	500	26
CS-MMT-PES	17.8	87.1%, Reactive black 5	500	27
QPEI-PES	12.8	~95%, Reactive black 5	500	28
GO-PSBMA-PES	11.9	99.2%, Reactive black 5<br>97.2%, Reactive red 49	500	29
HNTs-PIL-PES	11.8	94-96%, Reactive black 5	-	30
mHT-PES	6.3	95%, Reactive black 5<br>90%, Reactive red 49	1000	31

Supplementary Table 6. Metal ion leakage during filtration (30 mL filtrated DI water).

Membrane type	Detected metal ion concentration	Limiting metal ion concentration
MOPM-Fe^{3+}	0.0047 ppm, Fe^{3+}	0.3 ppm[a]; 0.3 ppm[b]; 0.3 ppm[c]
MOPM-Ag^{+}	0.0313 ppm, Ag^{+}	0.05 ppm[a]; 0.1 ppm[b]; 0.1 ppm[c]
MOPM-Zn^{2+}	not detected, Zn^{2+}	1.0 ppm[a]; 3.0 ppm[b]; 5.0 ppm[c]
MOPM-Ni^{2+}	not detected, Ni^{2+}	0.02 ppm[a]; 0.02 ppm[b]
MOPM-Zr^{4+}	not detected, Zr^{4+}	N/A

[a] Drinking-water quality standard (GB5749-2006), (2007, China).

[b] Guidelines for drinking-water quality 4thed, (2011, World Health Organization, WHO).

[c] National Secondary Drinking Water Regulations, (2007, USA).

Supplementary Table 7. Summary of PA concentration (mg/mL), metal salt content (mg/mL), assembly time (min) and pH value of assembly solution for fabricating MOPMs. The solute content during assembly was calculated based on solution volume of 30 mL.

Membrane name	PA concentration (mg/mL)	Metal salt type	Metal salt content (mg/mL)	Mole ratio (PA:M)	Assembly time (min)	pH value
MOPM-Ag^{+}	0.015	$AgNO_3$	2.8	1:7	60	2.22
MOPM-Zn^{2+}	0.015	$ZnCl_2$	2.3	1:7	60	2.20
MOPM-Ni^{2+}	0.015	$NiCl_2·6H_2O$	3.9	1:7	60	2.24
MOPM-Fe^{3+}	0.015	$FeCl_3·6H_2O$	4.2	1:7	60	2.21
MOPM-Zr^{4+}	0.015	$Zr(NO_3)_4·5H_2O$	7.0	1:7	60	2.23
**Condition experiment of MOPM-Fe^{3+}**						
MOPM-Fe^{3+}-1	0.015	$FeCl_3·6H_2O$	0.3	1:0.5	60	2.23
MOPM-Fe^{3+}-2	0.015	$FeCl_3·6H_2O$	0.6	1:1	60	2.21
MOPM-Fe^{3+}-3	0.015	$FeCl_3·6H_2O$	1.2	1:2	60	2.24
MOPM-Fe^{3+}-4	0.015	$FeCl_3·6H_2O$	3.0	1:5	60	2.22
MOPM-Fe^{3+}-5	0.015	$FeCl_3·6H_2O$	4.2	1:7	60	2.20
MOPM-Fe^{3+}-6	0.030	$FeCl_3·6H_2O$	8.4	1:7	60	2.14
MOPM-Fe^{3+}-7	0.045	$FeCl_3·6H_2O$	12.6	1:7	60	2.19
MOPM-Fe^{3+}-8	0.015	$FeCl_3·6H_2O$	6.3	1:10	60	2.24
MOPM-Fe^{3+}-9	0.015	$FeCl_3·6H_2O$	4.2	1:7	40	2.25
MOPM-Fe^{3+}-10	0.015	$FeCl_3·6H_2O$	4.2	1:7	50	2.22
MOPM-Fe^{3+}-11	0.015	$FeCl_3·6H_2O$	4.2	1:7	70	2.21
MOPM-Fe^{3+}-12	0.015	$FeCl_3·6H_2O$	4.2	1:7	80	2.20

Supplementary References

1. Cummings, C. Y. et al. Facile cation electro-insertion into layer-by-layer assembled iron phytate films. *Electrochem. Commun.* **12**, 1722-1726 (2010).

2. You, X. et al. Precise nanopore tuning for a high-throughput desalination membrane via co-deposition of dopamine and multifunctional poss. *J. Mater. Chem. A* **6**, 13191-13202 (2018).

3. Bi, R. et al. Graphene quantum dots engineered nanofiltration membrane for ultrafast molecular separation. *J. Membr. Sci.* **572**, 504-511 (2019).

4. Zhao, X. et al. Engineering amphiphilic membrane surfaces based on peo and pdms segments for improved antifouling performances. *J. Membr. Sci.* **450**, 111-123 (2014).

5. Cui, X. et al. Microstructure and corrosion resistance of phytic acid conversion coatings for magnesium alloy. *Appl. Surf. Sci.* **255**, 2098-2103 (2008).

6. Donnan, F. G. & Rose, R. C. Osmotic pressure, molecular weight, and viscosity of sodium alginate. *Can. J. Res.* **28**, 105-113 (2011).

7. Hernick, M. & Fierke, C. Mechanisms of metal-dependent hydrolases in metabolism. *Comprehensive Natural Products II*, 547-581 (2010).

8. Lee, K. P., Zheng, J., Bargeman, G., Kemperman, A. J. B. & Benes, N. E. PH stable thin film composite polyamine nanofiltration membranes by interfacial polymerisation. *J. Membr. Sci.* **478**, 75-84 (2015).

9. Wu, D., Huang, Y., Yu, S., Lawless, D. & Feng, X. Thin film composite nanofiltration membranes assembled layer-by-layer via interfacial polymerization from polyethylenimine and trimesoyl chloride. *J. Membr. Sci.* **472**, 141-153 (2014).

10. Mahdavi, H., Razmi, F. & Shahalizade, T. Polyurethane tfc nanofiltration membranes based on interfacial polymerization of poly(bis-mpa) and mdi on the polyethersulfone support. *Sep. Purif. Technol.* **162**, 37-44 (2016).

11. Wu, M. et al. Fabrication of composite nanofiltration membrane by incorporating attapulgite nanorods during interfacial polymerization for high water flux and antifouling property. *J. Membr. Sci.* **544**, 79-87 (2017).

12. Yan, F. et al. Improving the water permeability and antifouling property of thin-film composite polyamide nanofiltration membrane by modifying the active layer with triethanolamine. *J. Membr. Sci.* **513**, 108-116 (2016).

13. Soyekwo, F. et al. Cellulose nanofiber intermediary to fabricate highly-permeable ultrathin nanofiltration

membranes for fast water purification. *J. Membr. Sci.* **524**, 174-185 (2017).

14. Zhang, X., Lv, Y., Yang, H. C., Du, Y. & Xu, Z. K. Polyphenol coating as an interlayer for thin-film composite membranes with enhanced nanofiltration performance. *ACS Appl. Mater. Inter.* **8**, 32512-32519 (2016).

15. Zeng, Y., Wang, L., Zhang, L. & Yu, J. Q. An acid resistant nanofiltration membrane prepared from a precursor of poly(s-triazine-amine) by interfacial polymerization. *J. Membr. Sci.* **546**, 225-233 (2017).

16. Shan, L., Gu, J., Fan, H., Ji, S. & Zhang, G. Micro-phase diffusion-controlled interfacial polymerization for an ultrahigh permeability nanofiltration membrane. *ACS Appl. Mater. Inter.* **9**, 44820-44827 (2017).

17. Tan, Z., Chen, S. F., Peng, X. S., Zhang, L. & Gao, C. J. Polyamide membranes with nanoscale turing structures for water purification. *Science* **360**, 518-521 (2018).

18. Santanu, K., Jiang, Z. & Livingston, A. G. Sub-10 nm polyamide nanofilms with ultrafast solvent transport for molecular separation. *Science* **348**, 1347-1352 (2015).

19. Yang, L. B., Wang, Z. & Zhang, J. L. Highly permeable zeolite imidazolate framework composite membranes fabricated via a chelation-assisted interfacial reaction. *J. Mater. Chem. A* **5**, 15342-15355 (2017).

20. You, F., Xu, Y., Yang, X., Zhang, Y. & Shao, L. Bio-inspired ni (2+)-polyphenol hydrophilic network to achieve unconventional high-flux nanofiltration membranes for environmental remediation. *Chem. Commun.* **53**, 6128-6131 (2017).

21. Li, Y. et al. Antifouling, high-flux nanofiltration membranes enabled by dual functional polydopamine. *ACS Appl. Mater. Inter.* **6**, 5548-5557 (2014).

22. Fan, L. et al. Green coating by coordination of tannic acid and iron ions for antioxidant nanofiltration membranes. *RSC Adv.* **5**, 107777-107784 (2015).

23. Zhang, R. et al. Coordination-driven in situ self-assembly strategy for the preparation of metal-organic framework hybrid membranes. *Angew. Chem. Int. Ed.* **53**, 9775-9779 (2014).

24. Wang, J. et al. High flux electroneutral loose nanofiltration membranes based on rapid deposition of polydopamine/polyethyleneimine. *J. Mater. Chem. A* **5**, 14847-14857 (2017).

25. Zhao, S. & Wang, Z. A loose nano-filtration membrane prepared by coating hpan uf membrane with modified pei for dye reuse and desalination. *J. Membr. Sci.* **524**, 214-224 (2017).

26. Zhu, J. Y. et al. Elevated salt transport of antimicrobial loose nanofiltration membranes enabled by copper nanoparticles via fast bioinspired deposition. *J. Mater. Chem. A* **4**, 13211-13222 (2016).

27. Zhu, J., Tian, M., Zhang, Y., Zhang, H. & Liu, J. Fabrication of a novel "loose" nanofiltration membrane by facile blending with chitosan–montmorillonite nanosheets for dyes purification. *Chem. Eng. J.* **265**, 184-193

(2015).

28. Zhu, J., Zhang, Y., Tian, M. & Liu, J. Fabrication of a mixed matrix membrane with in situ synthesized quaternized polyethylenimine nanoparticles for dye purification and reuse. *ACS Sustain. Chem. Eng.* **3**, 690-701 (2015).

29. Zhu, J. et al. Surface zwitterionic functionalized graphene oxide for a novel loose nanofiltration membrane. *J. Mater. Chem. A* **4**, 1980-1990 (2015).

30. Yu, L., Zhang, Y., Zhang, H. & Liu, J. Development of a molecular separation membrane for efficient separation of low-molecular-weight organics and salts. *Desalination* **359**, 176-185 (2015).

31. Yu, L., Deng, J., Wang, H., Liu, J. & Zhang, Y. Improved salts transportation of a positively charged loose nanofiltration membrane by introduction of poly(ionic liquid) functionalized hydrotalcite nanosheets. *ACS Sustain. Chem. Eng.* **4**, 3292-3304 (2016).
